# Oxygen Vacancy-Rich 2D TiO_2_ Nanosheets: A Bridge Toward High Stability and Rapid Hydrogen Storage Kinetics of Nano-Confined MgH_2_

**DOI:** 10.1007/s40820-022-00891-9

**Published:** 2022-07-15

**Authors:** Li Ren, Wen Zhu, Yinghui Li, Xi Lin, Hao Xu, Fengzhan Sun, Chong Lu, Jianxin Zou

**Affiliations:** 1 National Engineering Research Center of Light Alloys Net Forming & State Key Laboratory of Metal Matrix Composites, Shanghai Jiao Tong University, Shanghai, 200240 People’s Republic of China; 2 Shanghai Engineering Research Center of Mg Materials and Applications & School of Materials Science and Engineering, Shanghai Jiao Tong University, Shanghai, 200240 People’s Republic of China; 3 Center of Hydrogen Science, Shanghai Jiao Tong University, Shanghai, 200240 People’s Republic of China; 4 Instrumental Analysis Center of SJTU, Shanghai Jiao Tong University, Shanghai, 200240 People’s Republic of China

**Keywords:** Hydrogen storage, MgH_2_, TiO_2_ nanosheets, Oxygen vacancies, Nanoconfinement

## Abstract

MgH_2_ has attracted intensive interests as one of the most promising hydrogen storage materials. Nevertheless, the high desorption temperature, sluggish kinetics, and rapid capacity decay hamper its commercial application. Herein, 2D TiO_2_ nanosheets with abundant oxygen vacancies are used to fabricate a flower-like MgH_2_/TiO_2_ heterostructure with enhanced hydrogen storage performances. Particularly, the onset hydrogen desorption temperature of the MgH_2_/TiO_2_ heterostructure is lowered down to 180 °C (295 °C for blank MgH_2_). The initial desorption rate of MgH_2_/TiO_2_ reaches 2.116 wt% min^−1^ at 300 °C, 35 times of the blank MgH_2_ under the same conditions. Moreover, the capacity retention is as high as 98.5% after 100 cycles at 300 °C, remarkably higher than those of the previously reported MgH_2_-TiO_2_ composites. Both in situ HRTEM observations and ex situ XPS analyses confirm that the synergistic effects from multi-valance of Ti species, accelerated electron transportation caused by oxygen vacancies, formation of catalytic Mg-Ti oxides, and stabilized MgH_2_ NPs confined by TiO_2_ nanosheets contribute to the high stability and kinetically accelerated hydrogen storage performances of the composite. The strategy of using 2D substrates with abundant defects to support nano-sized energy storage materials to build heterostructure is therefore promising for the design of high-performance energy materials.

**Supplementary Information:**

The online version contains supplementary material available at 10.1007/s40820-022-00891-9.

## Highlights

- A MgH_2_/TiO_2_ heterostructure with nano MgH_2_ assembled on oxygen vacancy-rich 2D TiO_2_ nanosheets was successfully fabricated via a simple solvothermal strategy.
- The MgH_2_/TiO_2_ heterostructure shows rapid desorption kinetics, low dehydrogenation temperature, and excellent cycling stability.
- In situ HRTEM observations and ex situ XPS analyses reveal that multi-valance of Ti species, presence of abundant oxygen vacancies, formation of catalytic Mg-Ti oxides, and confinement of TiO_2_ nanosheets, contribute to the high stability and kinetically accelerated hydrogen sorption performances of Mg.

**Supplementary Information:**

The online version contains supplementary material available at 10.1007/s40820-022-00891-9.

## Graphical abstract

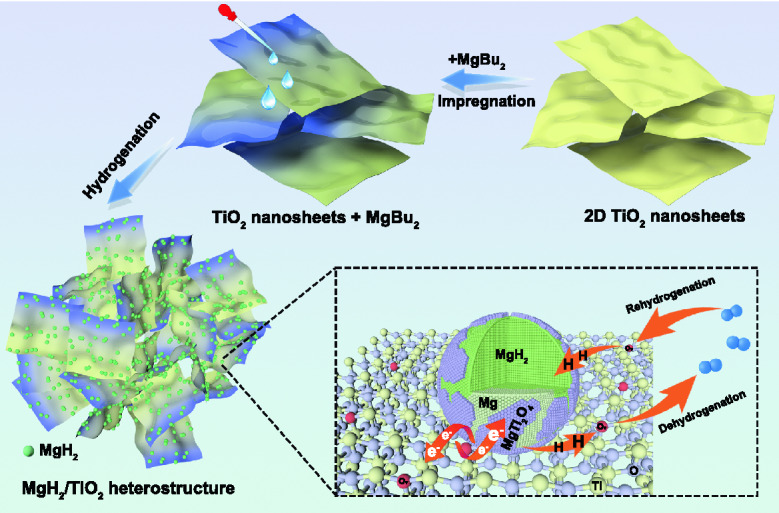

## Introduction

With the ever-increasing global energy demands and environmental concerns, clean and renewable energy is urgently demanded worldwide. Hydrogen has been considered as one of the most promising candidates to alternate fossil fuels, due to its intrinsic clean nature, environmental-friendliness, and high gravimetric energy density (142 MJ kg^−1^) [1–4]. However, an effective strategy to store hydrogen is essential to realizing the "hydrogen economy" [5, 6]. Compared with hydrogen storage in pressurized tanks or cryogenic containers, solid-state hydrogen storage methods potentially have the advantages of high safety, low cost, and exceptional hydrogen storage capacity [7]. Among various solid-state hydrogen storage materials, magnesium hydride (MgH_2_) has attracted tremendous attention, due to its high gravimetric (~ 7.6 wt% H_2_) and volumetric (~ 110 kg m^−3^ H_2_) hydrogen storage density, excellent reversibility, low cost, and non-toxicity [8, 9]. Unfortunately, the industrial application of MgH_2_ has been seriously hampered by its high operating temperature and slow de/re-hydrogenation kinetics. Particularly, a high temperature of at least 280 °C is required at 1 bar for desorption of coarse-grained MgH_2_, which far exceeds the working temperature of the proton exchange membrane fuel cell (PEMFC). Consequently, it is essential to improve the hydrogen storage performances of MgH_2_ for possible applications in the hydrogen energy field.

In the last few decades, extensive researches have been executed toward fabricating novel Mg-based hydrogen storage materials with enhanced performances, such as adding catalysts [10–13], alloying [14–16], and reducing the particle size of MgH_2_ [17–19]. From the extrinsic perspective, the use of additives to weaken the Mg-H bonding and promote the diffusion rate of hydrogen atoms, thus reducing the dehydrogenation temperature and accelerating the desorption rates, is regarded as an efficient strategy for enhancing the hydrogen desorption properties of MgH_2_. These additives include transition metals (TM) [20, 21], transition metal oxides [22, 23], nitrides [24, 25], halides [26, 27], and hydrides [28, 29]. Among them, transition metals (Ti, V, Nb, Ni, Co) and their compounds (TiO_2_, Nb_2_O_5_, V_2_O_3_) strike more attentions and have been proven to be the most effective additives to ameliorate the hydrogen sorption performances of MgH_2_, since transition metals with multiple valences favor the electron transfer during the de/re-hydrogenation processes [26]. The Fermi level of transition metals is located around s-type orbitals, which is a necessary condition to promote hydrogen dissociation and recombination [30]. Cui et al. [21] systematically investigated the catalytic mechanism of TM on the MgH_2_ by the synthesis of core (Mg)-shell (TM) structure. The results show that Mg-Ti composites, which can release hydrogen even under 200 °C, exhibit optimal desorption performances, owing to the lower electronegativity of Ti. Notably, it has been proved that TiO_2_ possesses a superior catalytic effect than metallic Ti [31]. Furthermore, TiO_2_ has many advantages, such as high natural abundance, low cost, and nontoxicity. Though the addition of catalysts through simple ball milling method can indeed ameliorate the kinetics of MgH_2_ to some extent, the improvement is restricted by the inevitable self-aggregation and growth of the additives during cycling, as well as the low density of exposed active sites.

On the other hand, from an intrinsic point of view, nano-sized MgH_2_, which owes the shortened diffusion path for hydrogen atoms and increased active sites for re/de-hydrogenation, has been successfully synthesized through various nanotechnologies, giving rise to improved hydrogen storage performances. For instance, the nano-confinement method has been proved to be a facile strategy to simultaneously improve the thermodynamics and kinetics of MgH_2_ while reducing the capacity loss as little as possible. Nano-sized MgH_2_ confined into porous carbon has demonstrated a noticeable decrease in its initial desorption temperature (~ 90 °C) compared with its bulk counterpart [32]. Yu et al. [33] synthesized Ni nanocrystal-decorated MgH_2_ nanoparticles anchored on graphene sheets that exhibit excellent hydrogen sorption properties. The composites absorb 5.4 wt% H_2_ within 10 min at 200 °C, and identical capacity can be released at the same temperature within 150 min. It is noted, however, that neither graphene nor porous carbon possesses good catalytic activities, and additional catalysts are needed to further improve hydrogen storage performances of MgH_2_. Although huge achievements have been achieved, it is still far from enough to enhance the hydrogen storage performances of MgH_2_ by solely depending on any methods mentioned above. In this respect, it is essential to fabricate MgH_2_ with extraordinary hydrogen storage performances by taking full advantage of both the intrinsic (nano-size) and the extrinsic (catalyst addition) strategy.

Recently, graphene-like two-dimensional (2D) transition metal oxides have been extensively studied due to their potential in energy-related applications with high surface area and unique electronic/thermal properties [34–37]. However, preparing hydrogen storage composites composed of graphene-like 2D TiO_2_ nanosheets (TiO_2_ NS) and MgH_2_ by a simple ball milling method would cause irreversible agglomeration of TiO_2_ NS, leading to seriously reduced catalytic active sites and deterioration of various spectacular intrinsic properties of 2D materials [38]. Thus, the idea of fabricating MgH_2_ (0D)/TiO_2_ (2D) heterostructure using graphene-like 2D TiO_2_ NS is triggered as it can not only facilitate the formation of nano-sized MgH_2_ through the confinement effect of TiO_2_ NS but also possess catalytic effect for MgH_2_. More importantly, the construction of MgH_2_/TiO_2_ heterostructure can effectively inhibit the self-stacking of TiO_2_ layers, leading to increased surface catalytic sites for MgH_2_. Additionally, defect engineering has also aroused more attention to adjusting the band structure and providing more catalytic active sites [39]. Among them, oxygen vacancy-rich TiO_2_ has been proved to remarkably promote electron transportation, enhance electrical conductivity, and provide more active sites for the capture and diffusion of hydrogen [40, 41].

In light of the above consideration, herein, taking full advantage of the intrinsic (nano-size) and extrinsic (catalyst addition) effects, we report a first attempt to design a novel self-assembled MgH_2_/TiO_2_ heterostructure with remarkably enhanced hydrogen sorption properties through facile solvothermal approach, where 2D TiO_2_ NS exhibit both nano-confinement and catalytic effects. Surprisingly, we found that the oxygen vacancies were introduced into TiO_2_ NS in the process of solvothermal treatment. Owing to the presence of oxygen vacancies and unique heterostructure, the obtained MgH_2_/TiO_2_ heterostructure exhibits impressive hydrogen storage performances with high stability and accelerated kinetics. Specifically, the 60MgH_2_/TiO_2_ heterostructure presents low onset desorption temperature (180 °C) and superior long-term cycling stability with a capacity retention of 98.5% after 100 cycles at 300 °C, remarkably higher than that of the state-of-the-art TiO_2_ catalyzed MgH_2_ systems.

## Experimental Section

### Synthesis of MgH_2_/TiO_2_ Heterostructure

#### Synthesis of Graphene-like 2D TiO_2_ Nanosheets

TiO_2_ nanosheets were synthesized by a surfactant self-assembly method [34]. Firstly, 1.48 mL titanium isopropoxide (TTIP, 99.9%, Macklin) was added to 2.1 mL concentrated HCl under vigorous stirring (solution A). Meanwhile, 0.4 g poly(ethylene oxide)-block-poly(propylene oxide)-block-poly(ethylene oxide) triblock copolymer (P123, Mn = 5,800) was dissolved in 8 mL ethanol (solution B) by ultrasonication. Afterward, solution B was added dropwise into solution A and kept stirring for 30 min at room temperature. Then, 5 mL of the above resultants with 40 mL ethylene glycol was then transferred into a 100 mL stainless steel autoclave and heated at 150 °C for 20 h. Finally, the products were centrifuged and washed with ethanol, followed by freeze-drying to obtain 2D TiO_2_ NS. Moreover, to improve the crystallinity and remove the impurities in TiO_2_ NS, the pristine TiO_2_ NS were annealed at different temperatures (300, 400, and 500 °C) for 2 h in the air (denoted as TiO_2_-T, T represents the annealing temperature).

#### Synthesis of the MgH_2_/TiO_2_ Heterostructure

MgH_2_/TiO_2_ heterostructure was prepared by a bottom-up solvothermal strategy of in situ growth of MgH_2_ nanoparticles on TiO_2_ substrates. *Di*-*n*-butyl-magnesium (MgBu_2_, 1 M solution in hexane, Sigma-Aldrich) and cyclohexane (C_6_H_12_, 99.5%, extra Dry, Sigma-Aldrich) were used as received states without further treatment. As provided in Table S1, different loading capacities of MgH_2_ on TiO_2_ NS of 40, 50, 60, 70, and 80 wt% were synthesized by changing the ratio between MgBu_2_ and TiO_2_ NS, which were denoted as 40MgH_2_/TiO_2_, 50MgH_2_/TiO_2_, 60MgH_2_/TiO_2_, 70MgH_2_/TiO_2_, and 80MgH_2_/TiO_2_, respectively. In a typical synthesis of 60MgH_2_/TiO_2_, 30 mg as-prepared TiO_2_-300 NS and 1.8 mL MgBu_2_ were firstly dispersed in 40 mL cyclohexane under sonication. The mixture was then transferred into a 100 mL custom-designed autoclave, filled with 4.5 MPa H_2_ pressure, and heated at 200 °C for 12 h under vigorous magnetic stirring [19, 42]. Finally, the resultants (named 60MgH_2_/TiO_2_ heterostructure) were collected by centrifugation, washed with cyclohexane several times, and dried at 80 °C overnight under dynamic vacuum. Moreover, blank MgH_2_ without TiO_2_ NS were also prepared under the same conditions for comparison.

### Characterization and Measurements

The phase composition was characterized by using X-ray diffraction (XRD, Mini Flex 600) with Cu K_α_ radiation operated at 40 kV and 15 mA. The samples were prepared in an argon-filled glovebox and sealed in a custom-designed holder covered by the Scotch tape to avoid the possible oxidation during the XRD tests. The microstructure and morphological analyses were performed by scanning electron microscope (SEM, MIRA3 LHM) and transmission electron microscope (TEM, FEI Talos F200X G2). For TEM observations, the preparation of samples was carried out in the glovebox. The samples were dispersed in cyclohexane, sonicated, dropped on a copper grid, and rapidly transferred to the equipment. The preparation conditions of samples for SEM tests were similar to those for the TEM observation, except that the samples were dispersed in cyclohexane and dropped onto the silicon wafer. The in situ high-resolution transmission electron microscope (HRTEM) observation was applied to explore the microstructural evolution during the decomposition of the MgH_2_/TiO_2_ heterostructure under an irradiation beam current of 3 nA [43]. The valence state and chemical bonding nature of constituent elements of MgH_2_/TiO_2_ composites were analyzed by X-ray photoelectron spectroscopy (XPS, Kratos AXIS Ultra DLD). For XPS analyses, a special air-proof transfer vessel was used to transfer the samples from glovebox to the equipment. Electron paramagnetic resonance (EPR) measurement was performed at room temperature using a Bruker spectrometer (EMXplus-9.5/12). Solid-state ^1^H magic-angle spinning nuclear magnetic resonance (MAS NMR) was carried out on a Bruker AVANCE NEO 600 spectrometer. Brunner–Emmet–Teller (BET) surface areas of all samples were measured by the N_2_ isothermal ad/de-sorption tests at 77 K on Autosorb-IQ3 apparatus. Differential scanning calorimetry (DSC, NETZSCH STA 449 F3) measurements were performed at different heating rates (3, 5, and 10 °C min^−1^) under a flowing Ar atmosphere to analyze the desorption kinetic properties. The DSC data were further processed by the Kissinger method to figure out the desorption apparent activation energy (*E*_*a*_) according to the following equation:

1 $$\mathrm{ln}\left(\frac{\beta }{{T}_{p}^{2}}\right)=-\frac{{E}_{a}}{R{T}_{p}}+C$$

where *β* represents the heating rate, *T*_*p*_ refers to the peak temperature, R is the universal gas constant (R = 8.314 J mol^−1^ K^−1^), and C is a constant.

The isothermal ab/de-sorption tests and temperature program desorption (TPD) measurements of samples were carried out by a Sievert-type pressure-composition-temperature apparatus (PCT, Shanghai Institute of Microsystem and Information Technology). For the isothermal ab/de-sorption tests, samples were loaded into the vessel in the glove box and were heated to the preset temperature at a heating rate of 10 °C min^−1^. The initial hydrogen pressures for isothermal ab/de-sorption were 3 and 0.001 MPa, respectively. Besides, the hydrogenated samples were heated from room temperature to 350 °C with a heating rate of 2 °C min^−1^ to perform TPD measurements. The hydrogen pressure/duration/temperature parameters for cycling tests are set as 3 MPa/15 min/300 °C for re-hydrogenation and 0.001 MPa/15 min/300 °C for dehydrogenation. The hydrogen storage capacity was calculated in the weight percent of the entire composites including the TiO_2_ scaffold.

## Results and Discussion

### Synthesis and Characterizations of the MgH_2_/TiO_2_ Heterostructure

**Scheme 1:**
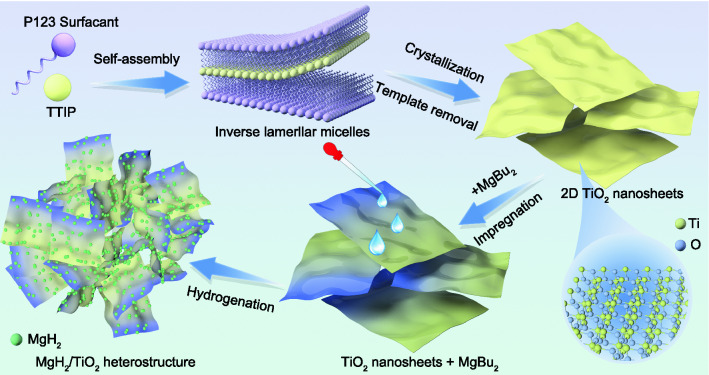
Synthesis process illustration of the MgH_2_/TiO_2_ heterostructure

The preparation procedure of the MgH_2_/TiO_2_ heterostructures is schematically illustrated in Scheme 1, including the synthesis of 2D TiO_2_ NS and subsequent impregnation of MgBu_2_ as well as the bottom-up self-assembly of MgH_2_ nanoparticles anchored on TiO_2_ NS. In a typical surfactant self-assembly synthesis process of TiO_2_ NS, P123 and ethylene glycol (EG) acted as co-surfactants which played the role of structure-directing agents to form inverse lamellar micelles. The precursor oligomers (TTIP) were then self-assembled into lamellar structures with surfactant molecules, inducing the formation of layered inorganic oligomer agglomerates. Afterward, solvothermal treatment was carried out to promote the organization and crystallization of 2D TiO_2_ NS. Subsequently, the TiO_2_ NS were ultrasonically dispersed into the C_6_H_12_ solution followed by the dropwise addition of MgBu_2_. Finally, the MgH_2_/TiO_2_ heterostructure was obtained in the above dispersed suspension by hydrogenation of MgBu_2_ on TiO_2_ NS through a facile solvothermal method.

**Fig. 1:**
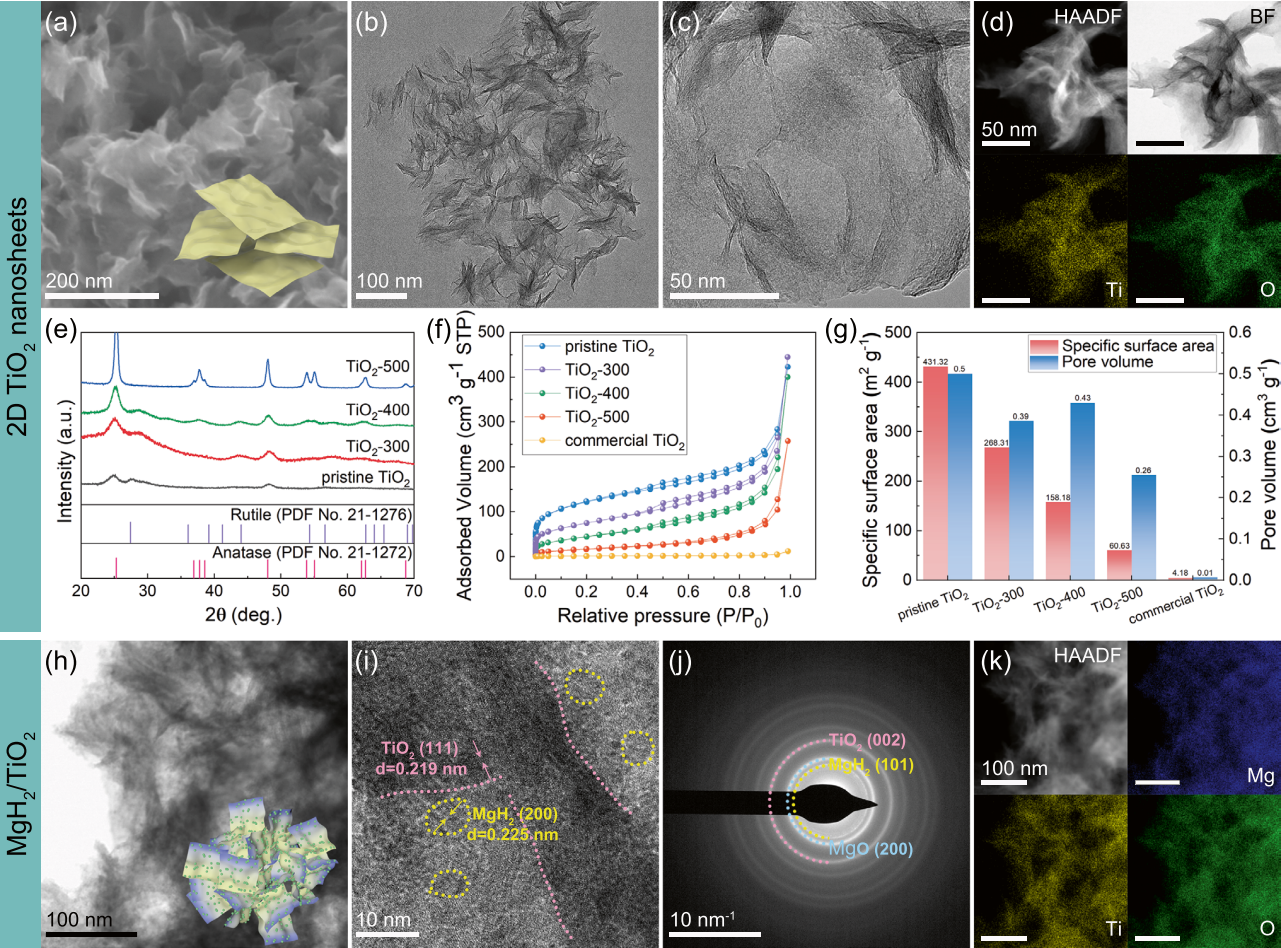
**a** Typical SEM image of TiO_2_ NS. **b, c** Typical TEM images at different scales. **d** Representative HAADF and BF images of TiO_2_ NS as well as corresponding EDS elemental mapping results. **e** XRD patterns of TiO_2_ NS. **f, g** N_2_ ad/de-sorption isotherms and corresponding specific surface areas and pore-size distribution of pristine TiO_2_ NS, commercial TiO_2_, and TiO_2_ NS treated at different annealing temperatures. **h-j** BF, HRTEM, and SAED images of as-synthesized 60MgH_2_/TiO_2_ heterostructure. **k** HAADF images of as-synthesized 60MgH_2_/TiO_2_ heterostructure and corresponding EDS elemental mapping results

The typical morphology and structural features of the ultrathin 2D TiO_2_ NS were observed using SEM and TEM. As shown in Fig. 1a–c, the pristine TiO_2_ NS exhibit a morphology of 2D corrugated ultrathin nanosheets with their edges roll up owing to the surface tension, which is also observed in graphene as a general phenomenon. The sizes of the TiO_2_ NS vary from 150 to 200 nm as seen from the TEM images. The thickness of the prepared TiO_2_ NS was further characterized using AFM and TEM (Fig. S1). The obtained TiO_2_ NS have a thickness of around 3.7 nm, corresponding to 5–6 monolayers, which confirms the formation of ultrathin nanosheets. In addition, the sheet-like structure of TiO_2_ can be clearly distinguished in the HAADF and BF images (Fig. 1d), which may play a morphology-directing role in the solution-phase synthesis of nanostructured MgH_2_. The EDS elemental mapping images demonstrate the homogeneous distribution of Ti and O elements (Fig. 1d).

Furthermore, the influence of the annealing temperature on the morphology of TiO_2_ NS was also taken into account (Fig. S2). It is worth emphasizing that TiO_2_-300 and TiO_2_-400 exhibit a similar sheet-like structure as the pristine TiO_2_ NS, except for the slight granulation after annealing at 400 °C. However, the crystallite size and crystallinity of TiO_2_-500 increase obviously and the 2D nanostructures can hardly be observed. This phenomenon coincides well with the previous report [44]. Moreover, we also explored the stability of TiO_2_ NS in the hydrogen atmosphere and it can be seen that the sheet-like structure of TiO_2_ is still preserved after treatment in H_2_ at 300 °C for 2 h (Fig. S2). Figure 1e shows the XRD patterns of pristine TiO_2_, TiO_2_-300, TiO_2_-400, and TiO_2_-500. According to the XRD patterns, pristine TiO_2_ NS are mainly composed of the anatase phase (JCPDS No. 21–1272), as well as a small amount of rutile phase (JCPDS No. 21–1276). After calcination treatment, the intensities of the diffraction peaks are much sharper and stronger, manifesting an increase in crystallinity, coinciding well with the SEM and TEM results (Fig. S2). Additionally, the diffraction peaks of rutile phase disappear and crystalline diffraction peaks assigned to anatase TiO_2_ crystal can be identified when the annealing temperature increases up to 400 and 500 °C. The specific surface area and pore size distribution of TiO_2_ NS were further investigated (Fig. 1f–g). Compared with commercial TiO_2_, the specific surface area of TiO_2_ NS is increased by 14–100 times, attributed to the special 2D sheet-like structures. The TiO_2_ NS possess a specific surface area of 431.32 m^2^ g^−1^, which is almost 100 times higher than that of the commercial TiO_2_ (~ 4 m^2^ g^−1^). Besides, it still possesses a high specific surface area of 268.31 m^2^ g^−1^ even after calcination treatment (300 °C). Thus, we selected TiO_2_-300 as the substrates to fabricate the MgH_2_/TiO_2_ heterostructure, and the TiO_2_ mentioned below all refers to TiO_2_-300 unless otherwise specified. The TiO_2_ NS with ultrahigh specific surface area will provide more nucleation sites for nanoparticles, which could be ingeniously employed as the excellent scaffold for the self-assembly of MgH_2_ NPs.

To fabricate MgH_2_/TiO_2_ heterostructure, MgBu_2_ was used as the precursor and added into TiO_2_ suspension followed by the solvothermal method under 4.5 MPa of hydrogen pressure. Impressively, the color of TiO_2_ suspension fortuitously changed from white to dark blue with the addition of MgBu_2_. The obvious blue color indicates the presence of oxygen vacancies caused by MgBu_2_ [39], which will be discussed in detail in the following section. Figure 1h shows the typical TEM images of as-synthesized 60MgH_2_/TiO_2_ heterostructure. It can be seen that TiO_2_ nanosheets still maintain a graphene-like structure after the self-assembly of MgH_2_ nanoparticles on them. Besides, 2D TiO_2_ nanosheets are automatically arranged into flower-like structures to support and encapsulate MgH_2_ nanoparticles, tremendously restricting the agglomeration of MgH_2_ NPs and simultaneously hampering the re-stacking of TiO_2_ NS in turn. According to the measurement of lattice spacing in HRTEM images (Fig. 1i), the interplanar spacings of 0.225 and 0.219 nm could be attributed to the (200) plane of MgH_2_ (JCPDS No. 12–0697) and (111) plane of TiO_2_ (JCPDS No. 21–1276), respectively. In addition, the diffraction rings in SAED pattern of 60MgH_2_/TiO_2_ (Fig. 1j) reveal the formation of polycrystalline structure. Furthermore, the 2D wrinkled sheet-like structure of TiO_2_ could be well distinguished from the HAADF images. EDS elemental mapping images confirm the homogeneous distribution of Mg, Ti, and O elements in the MgH_2_/TiO_2_ heterostructure (Fig. 1k).

### Improved Hydrogen Storage Properties of the MgH_2_/TiO_2_ Heterostructure

**Fig. 2:**
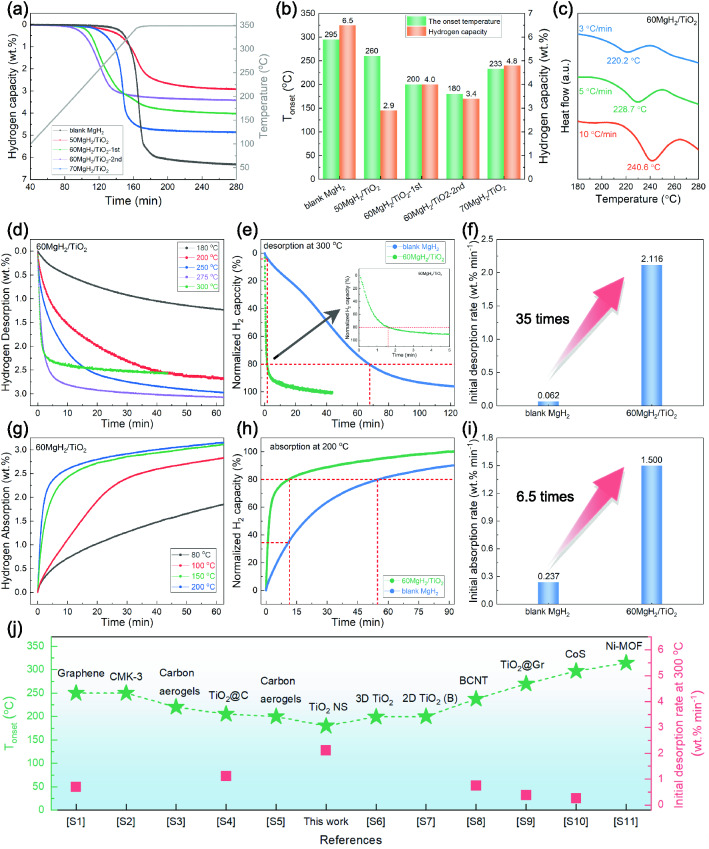
**a, b** TPD curves and corresponding onset desorption temperature and hydrogen capacity of blank MgH_2_, 50MgH_2_/TiO_2_, 60MgH_2_/TiO_2_-1st, 60MgH_2_/TiO_2_-2nd, and 70MgH_2_/TiO_2_. **c** DSC curves of 60MgH_2_/TiO_2_. **d** Isothermal dehydrogenation profiles of 60MgH_2_/TiO_2_. **e** Isothermal dehydrogenation curves and **f** initial desorption rate of 60MgH_2_/TiO_2_ and blank MgH_2_ at 300 °C. **g** Isothermal re-hydrogenation profiles of 60MgH_2_/TiO_2_. **h** Isothermal re-hydrogenation curves and **i** initial absorption rate of 60MgH_2_/TiO_2_ and blank MgH_2_ at 200 °C. **j** Comparison of the onset desorption temperature and initial desorption rates at 300 °C of MgH_2_/TiO_2_ heterostructure with those of other nano-confined MgH_2_ systems and TiO_2_ catalyzed MgH_2_ systems in the literature (Table S2)

The hydrogen storage performances of the MgH_2_/TiO_2_ heterostructure were further examined. Figure 2a shows the TPD profiles of blank MgH_2_ and the MgH_2_/TiO_2_ with different loading of MgH_2_ and Fig. 2b shows the comparison of the onset desorption temperature and hydrogen capacity of different samples. As the control sample, the blank MgH_2_ starts to desorb hydrogen at about 295 °C, which is higher than those of MgH_2_/TiO_2_ composites, indicating that the TiO_2_ NS indeed have a positive effect on the dehydrogenation of MgH_2_. Concerning the MgH_2_/TiO_2_ with different loading capacities of MgH_2_, the onset desorption temperatures decrease in the sequence of 50MgH_2_/TiO_2_ (260 °C), 70MgH_2_/TiO_2_ (233 °C), and 60MgH_2_/TiO_2_ (200 °C). Thus, we further explored the hydrogen storage performances of 60MgH_2_/TiO_2_, and the MgH_2_/TiO_2_ heterostructure mentioned below refers to the 60MgH_2_/TiO_2_ unless otherwise specified. In the first non-isothermal desorption process, the 60MgH_2_/TiO_2_ exhibits the initial desorption temperature of 200 °C with a total hydrogen release of 4 wt%. Notably, the 60MgH_2_/TiO_2_ composite possesses a lower onset desorption temperature of 180 °C along with a hydrogen capacity of 3.4 wt% in the second non-isothermal dehydrogenation process. The lower desorption temperature in the second cycle reveals that the catalytic activity of TiO_2_ NS varies upon de/ab-sorption behavior. The capacity loss after the first desorption may be attributed to the irreversible reaction between MgH_2_ and TiO_2_. It is therefore reasonable to speculate that the chemical environment of MgH_2_ and TiO_2_ in the first dehydrogenated sample should be considerably different from the as-synthesized sample, indicating the formation of catalytic species during initial hydrogen cycling, which is similar to what shown in previous reports [45–48], and will be discussed in later sections. In addition, DSC profiles (Figs. 2c and S3) show that the peak desorption temperature of commercial MgH_2_ is 408.6 °C under the heating rate of 3 °C min^−1^, while it tremendously reduces to 220.2 °C for the 60MgH_2_/TiO_2_ heterostructure. According to the fitting results of DSC by Kissinger’s equation, the apparent activation energy of 60MgH_2_/TiO_2_ is determined to be 106.7 kJ mol^−1^ H_2_, much lower than that of the commercial MgH_2_ (142.27 kJ mol^−1^ H_2_).

In order to further explore the influence of TiO_2_ NS on MgH_2_, the isotherm de/ab-sorption measurements were carried out for the 60MgH_2_/TiO_2_ heterostructure as well as control samples (blank MgH_2_ and commercial MgH_2_). Figure 2d shows the isotherm desorption curves of the 60MgH_2_/TiO_2_. As can been seen, the 60MgH_2_/TiO_2_ releases hydrogen of 0.51, 1.56, 2.14, 2.83, and 2.43 wt% at the temperature of 180, 200, 250, 275, and 300 °C within 10 min, respectively. Moreover, even at a lower temperature of 180 °C, the composite can still desorb 1.24 wt% of hydrogen within 60 min, corresponding to 41% of the maximum hydrogen storage capacity. At 250 °C, 2.14 wt% of hydrogen can be desorbed within 10 min, while no hydrogen desorption is detected for the blank MgH_2_ and commercial MgH_2_ under identical conditions (Fig. S4). In addition, blank MgH_2_ needs 68 min to release 80% of its theoretical hydrogen capacity at 300 °C. In contrast, it takes only 1.8 min for the 60MgH_2_/TiO_2_ composite to reach the identical reaction extent (Fig. 2e). Impressively, the initial desorption rate is increased by 35 times for the 60MgH_2_/TiO_2_ (Fig. 2f) when compared to that of blank MgH_2_. This demonstrates the remarkably improved hydrogen storage performances of MgH_2_ through the introduction of TiO_2_ NS.

The isothermal re-hydrogenation curves of samples are presented in Fig. 2g-i. The 60MgH_2_/TiO_2_ could absorb hydrogen even at a temperature as low as 80 °C with a hydrogen capacity of 1.86 wt% within 60 min. Such a low hydrogenation temperature meets the target of working temperature set by DOE for light-duty vehicle applications [7]. A hydrogen capacity of 2.65 wt%, corresponding to 80% of the maximum hydrogen capacity, is achieved at 200 °C within 11.9 min for the 60MgH_2_/TiO_2_ heterostructure. However, it takes a long time of 54.8 min for the blank MgH_2_ to achieve the same reaction extent (Fig. 2h). Additionally, it is noteworthy that the initial absorption rate of 60MgH_2_/TiO_2_ at 200 °C is 6.5 times that of the blank MgH_2_ (Fig. 2i). The superior hydrogen desorption performances, such as high initial desorption rate and low desorption temperature of the 60MgH_2_/TiO_2_, remarkably exceed those of the state-of-the-art MgH_2_ systems reported in literature (Fig. 2j and Table S2).

**Fig. 3:**
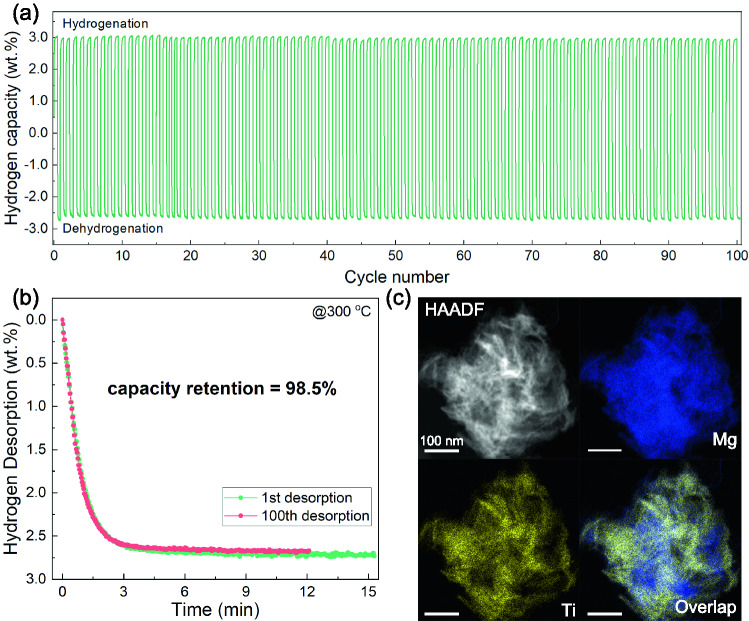
**a** Reversible hydrogen absorption and desorption cycling profiles of 60MgH_2_/TiO_2_ at 300 °C. **b** Hydrogen desorption curves of 60MgH_2_/TiO_2_ at different cycles (1st and 100th). **c** HAADF images of 60MgH_2_/TiO_2_ after 100 cycles and corresponding EDS elemental mapping results

Cycling stability is also a key factor for the practical application of hydrogen storage materials. To further evaluate the stability of the 60MgH_2_/TiO_2_, long-term (100 cycles) isothermal de/re-hydrogenation was tested at a relatively high temperature of 300 °C (Fig. 3a). It can be seen that MgH_2_/TiO_2_ shows negligible degradation of the capacity even after 100 cycles (from 2.71 to 2.67 wt%) and a capacity retention as high as 98.5% is achieved (Fig. 3b), which is highly comparable with and even surpasses those of the previously reported Mg-based hydrogen storage materials catalyzed by TiO_2_ and other transition metal oxides. Additionally, compared to the first cycle, the MgH_2_/TiO_2_ heterostructure exhibits a faster initial desorption rate during the second cycle (Fig. S5), which is associated with the formation of the catalytic oxide mentioned above. Moreover, the MgH_2_/TiO_2_ heterostructure still maintains flower-like structures and shows no obvious agglomeration of MgH_2_ and restacking of TiO_2_ NS even after 100 cycles (Fig. 3c). However, with the absence of TiO_2_ NS, blank MgH_2_ exhibits severe growth and agglomeration after several de/re-hydrogenation cycles (Fig. S6). The high stability of the MgH_2_/TiO_2_ heterostructure is presumably ascribed to the confinement effect of 2D TiO_2_ NS on MgH_2_ and robust interfacial contact between host and support materials.

Therefore, the MgH_2_/TiO_2_ heterostructure exhibits rapid hydrogen ab/de-sorption kinetics and excellent long-term cycling stability than those of blank MgH_2_, highlighting the crucial role of the 2D TiO_2_ nanosheets, which simultaneously possess catalytic effect to MgH_2_ and inhibit the agglomeration of MgH_2_.

### Catalytic Mechanism of the MgH_2_/TiO_2_ Heterostructure

**Fig. 4:**
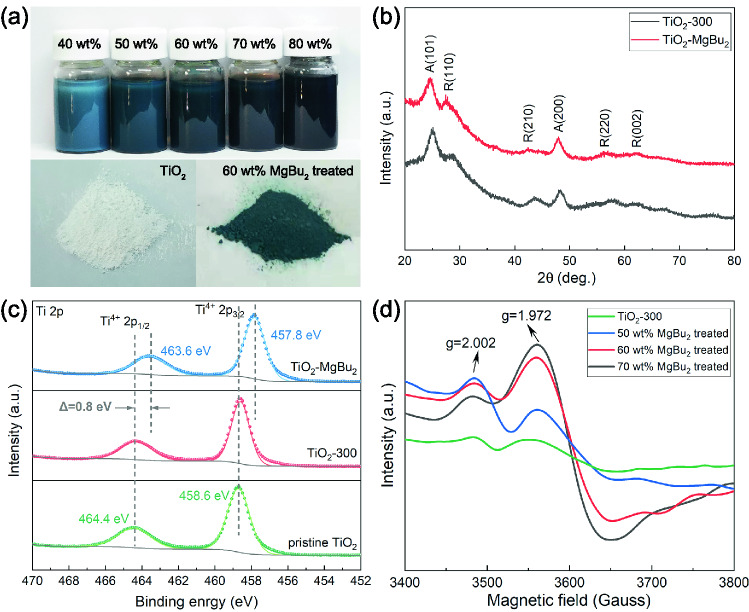
**a** Optical photographs of TiO_2_ NS suspension treated with different amounts of MgBu_2_. **b** XRD patterns of TiO_2_-300 and MgBu_2_-treated TiO_2_ NS. **c** High-resolution XPS spectra of Ti 2*p*. **d** EPR spectra of TiO_2_-300 and TiO_2_ NS treated with different amounts of MgBu_2_ ("40wt%/ 50wt%/ 60wt%/ 70wt%/ 80wt% " refers to the weight percentage of MgH_2_ in the MgH_2_/TiO_2_ heterostructure)

In order to further understand the mechanism of improved sorption performances of the MgH_2_/TiO_2_ heterostructure, we further characterized the phase components and microstructure of samples at different states. Figure 4 displays the optical photographs and microstructure of pristine TiO_2_ NS and MgBu_2_-treated TiO_2_ NS. As shown in Fig. 4a, the MgBu_2_-treated TiO_2_ NS exhibit in different colors ranging from blue to black, in marked contrast with the white pristine TiO_2_ powders. The color change of TiO_2_ is ascribed to the presence of oxygen vacancies, which coincides well with the previous work [39–41, 49]. Herein, we introduce oxygen vacancies into TiO_2_ NS by a facile strategy, that is, the impregnation of MgBu_2_ into TiO_2_ NS. We discover that the MgBu_2_ could not only act as the reducing agent to fabricate oxygen vacancy-rich blue TiO_2_ NS but also serve as the precursor of MgH_2_ to promote the self-assembly of MgH_2_ nanoparticles onto TiO_2_ NS through subsequent solvothermal approaches. Such a "one-stone-two-birds" effect enables the MgH_2_/TiO_2_ heterostructure as a promising hydrogen storage material with superior performances. Figure 4b displays the identical crystalline structure with the anatase phase in the majority and that of rutile in minority for different TiO_2_ samples, indicating that the impregnation of MgBu_2_ does not destroy the intrinsic crystal structure of the TiO_2_ nanosheets. Besides, XPS spectra of elemental Ti (Fig. 4c) and O (Fig. S7) are provided. The high-resolution XPS spectra of Ti 2*p* of the pristine TiO_2_ NS and TiO_2_-300 at 464.4 and 458.6 eV can be attributed to Ti^4+^ 2*p*_1/2_ and Ti^4+^ 2*p*_3/2_, respectively, while the peaks of TiO_2_ NS treated with MgBu_2_ slightly shift toward lower binding energy, indicating that Ti^3+^ is generated with the formation of oxygen vacancies. The O 1*s* peaks located at 531.8, 530.8, and 529.5 eV belong to adsorbed oxygen (O_A_), oxygen vacancy (O_V_), and lattice oxygen (O_L_) in the MgBu_2_-treated TiO_2_ NS, respectively (Fig. S7). Based on the peak area of O_V_ to the overall peak area of O 1*s*, the percentage of O_V_ on the surface of the MgBu_2_-treated TiO_2_ is determined to be 32.53%. Additionally, the concentration of oxygen vacancies on the surface of the as-synthesized MgH_2_/TiO_2_ heterostructure (33.4%) shows no significant change when compared to that on the TiO_2_ NS treated with MgBu_2_ (Fig. S7).

Moreover, EPR measurement was carried out to further explore the existence of oxygen vacancies in TiO_2_ NS treated with MgBu_2_. As shown in Fig. 4d, the TiO_2_ NS treated with MgBu_2_ exhibit typical EPR signals at about g = 1.972 and g = 2.002, which could be attributed to Ti^3+^ and oxygen vacancies in the lattice, respectively [40, 50]. All these results strongly prove that the oxygen vacancies have been successfully introduced into TiO_2_ nanosheets by the "one-stone-two-birds" strategy. Due to the higher electrical conductivity, numerous active sites, and enlarged lattice space derived from oxygen vacancies, the formation of oxygen vacancy-rich blue TiO_2_ could not only accelerate the migration of electrons and diffusion of hydrogen atoms but also provide more active sites for the capture of hydrogen and nucleation of MgH_2_/Mg. Thus, the MgH_2_/TiO_2_ heterostructure displays superior hydrogen ab/de-sorption kinetic properties.

**Fig. 5:**
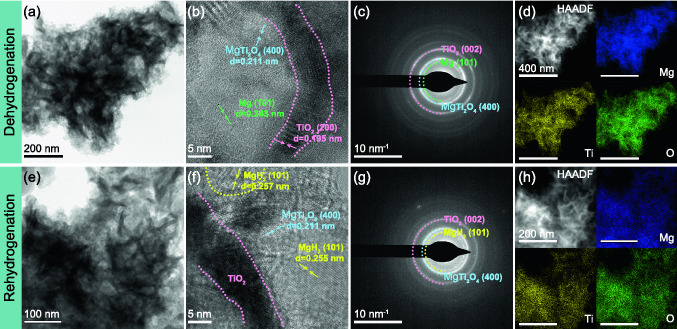
BF, HRTEM, SAED, HAADF images as well as EDS elemental mapping results of dehydrogenated **a-d** and re-hydrogenated **e–h** 60MgH_2_/TiO_2_ heterostructure

In order to elucidate the catalytic mechanism, the structural stability of the MgH_2_/TiO_2_ composites during the hydrogen de/ab-sorption process was further investigated. Figure 5 shows the TEM images of dehydrogenated and re-hydrogenated 60MgH_2_/TiO_2_ heterostructure. Surprisingly, there are no significant differences in morphology among the as-synthesized (Fig. 1h–k), dehydrogenated (Fig. 5a–d), and re-hydrogenated (Fig. 5e–h) samples, further confirming the thermal stability of the MgH_2_/TiO_2_ composites. Notably, by measuring the interplanar spacing of the well-resolved lattice fringes (Fig. 5b, f), we observe a new Mg-Ti ternary oxide, which is discussed in detail below. Thus, the establishment of the flower-like heterostructure has rendered the ability to well maintain the primary structure of the TiO_2_ scaffold and MgH_2_ nanoparticles, thereby improving the long-term cycling stability of MgH_2_ and facilitating rapid transport of electrons and hydrogen.

**Fig. 6:**
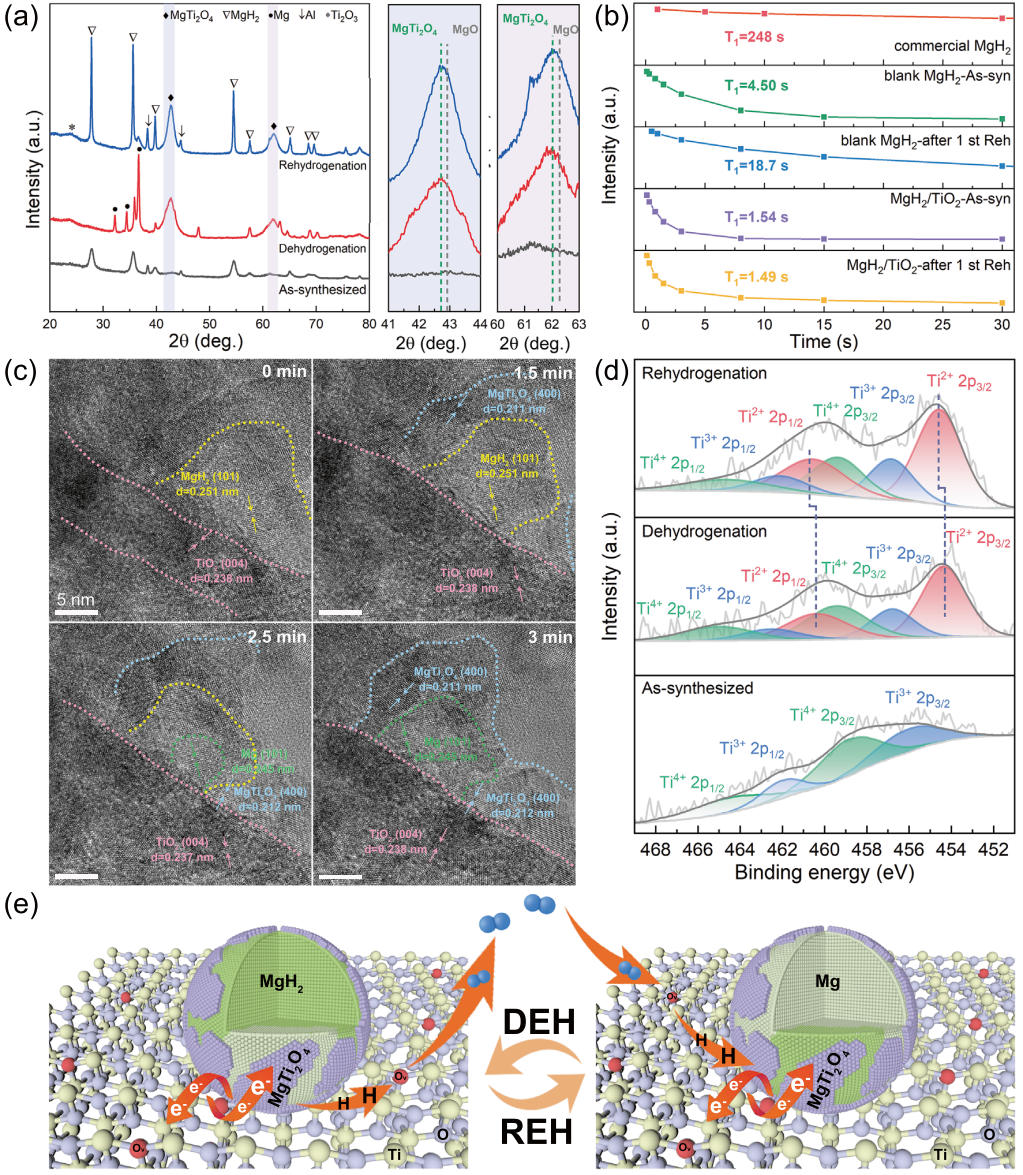
**a** XRD patterns of the as-synthesized, dehydrogenated, and re-hydrogenated 60MgH_2_/TiO_2_ heterostructure. **b** NMR spin lattice relaxation times of 60MgH_2_/TiO_2_, commercial MgH_2_, and blank MgH_2_. **c** In situ HRTEM images showing the microstructure evolution of the hydrogenated 60MgH_2_/TiO_2_ during the hydrogen desorption process. **d** High-resolution Ti 2*p* spectra of 60MgH_2_/TiO_2_ at different states. **e** Schematic diagram showing the hydrogenation and dehydrogenation mechanisms of MgH_2_/TiO_2_ heterostructure

We further explored the phase transformation and chemical valence state change of MgH_2_/TiO_2_ at different states. The XRD patterns of as-synthesized samples exhibit broad diffraction peaks indicating the presence of nano-sized MgH_2_ (Fig. 6a). No peaks from MgO (which generally appear at the peak position of ~ 42.9° and 62.3° [11]) can be detected in the XRD pattern of as-synthesized MgH_2_/TiO_2_, indicating the absence of MgO. After dehydrogenation, in addition to the main phase of Mg, two new broad peaks located at 42.7° and 62.0° appear in the XRD patterns, which are close to the peak positions of MgO. In the subsequent hydrogen absorption process, Mg phase transforms to MgH_2_ and those two characteristic peaks assigned to Mg_2_TiO_4_ (JCPDS No. 16–0215) show no obvious change. Additionally, as shown in Fig. S8, the phase composition of the MgH_2_/TiO_2_ heterostructure remains unchanged after 100 de/re-hydrogenation cycles by showing very similar XRD patterns. Such a Mg-Ti oxide phase may possess a catalytic effect for the de/re-hydrogenation of MgH_2_ [45, 47]. As is well known, the existence of MgO will deteriorate the performance of MgH_2_; hence, higher temperature is required for the release of hydrogen [51]. However, the MgH_2_/TiO_2_ composites perform excellent hydrogen desorption properties. To further prove that these two peaks belong to the highly catalytic Mg-Ti oxide instead of less-catalytic MgO, ^1^H NMR test was conducted (Figs. 6b and S9). A shorter spin–lattice relaxation time of MgH_2_/TiO_2_ (~ 1.54 s) compared with that of blank MgH_2_ (~ 4.50 s) indicates a faster H motion in MgH_2_/TiO_2_. In particular, the MgH_2_/TiO_2_ heterostructure exhibits a much shorter spin–lattice relaxation time of 1.49 s after the 1st re-hydrogenation than that of the as-synthesized MgH_2_/TiO_2_, indicating that new species with catalytic activity have formed during the first dehydrogenation process. Thus, it is demonstrated that two characteristic peaks centered at 42.7° and 62.0° are assigned to the ternary Mg-Ti oxide phase (MgTi_2_O_4_), having much higher catalytic activity than MgO. It can be deduced that the Mg-Ti oxide is formed by the chemical reaction at the interface between MgH_2_ and TiO_2_ NS during the first hydrogen desorption process. The similar phenomenon, for which Ti/Nb oxide could react with MgH_2_ and result in the formation of a new ternary oxide, has also been reported in the previous work [48]. Compared to Mg/MgH_2_, the broader diffraction peaks of the ternary Mg-Ti oxide phase manifest its smaller crystallite size than that of Mg/MgH_2_.

To further clarify the role of the ternary Mg-Ti oxide in the dehydrogenation process, in situ HRTEM observations were carried out by real-time recording of the structure and composition evolution of hydrogenated MgH_2_/TiO_2_ during the hydrogen desorption process (Fig. 6c). Additional images with more details are shown in Fig. S10. Before electron beam irradiation (at 0 min), MgH_2_ (101) and TiO_2_ nanosheets could be identified according to the lattice fringe spacings. After irradiation under a beam current of 3 nA for 1.5 min, a portion of MgH_2_ react with TiO_2_, generating a thin Mg-Ti oxide layer. After 2.5 min of irradiation, the MgH_2_ near Mg-Ti oxide begins to release hydrogen to form Mg. The observed area releases hydrogen completely after 3 min. It can be seen that, unlike the neat MgO layer, the in-built ternary Mg-Ti oxide catalyst could act as a pathway to facilitate hydrogen diffusion, resulting in improved reaction kinetics. Through the pathways provided by Mg-Ti oxide, the hydrogen can diffuse into/out of Mg/MgH_2_, possibly by a formation of metastable titanium hydride (TiH_x_) species [19, 52], which coincides well with the XPS chemical state shifts discussed later. In addition, Mg-Ti oxide at the MgH_2_/TiO_2_ interfaces could also strongly anchor MgH_2_ nanoparticles, which significantly inhibits the growth and agglomeration of MgH_2_ particles during cycling. Moreover, the high ductility and flexibility of the Mg-Ti oxide [53] can significantly buffer the volume change of MgH_2_ particles during de/re-hydrogenation cycles, preventing the detachment of MgH_2_ from the TiO_2_ substrate and thereby ensuring the excellent cyclic stability.

Moreover, XPS analyses were employed to explore the evolution of the bonding state of Ti in the process of de/re-hydrogenation and the results are shown in Fig. 6d. XPS survey spectrum indicates the coexistence of Mg, O, and Ti elements (Fig. S11), well coincident with the EDS mapping results. As shown in the high-resolution Ti 2*p* XPS spectra, the signals of Ti^4+^ species (458.8 eV for 2*p*_3/2_ and 464.5 eV for 2*p*_1/2_) can be detected in all samples at different states, indicating that TiO_2_ nanosheets still exist in the MgH_2_/TiO_2_ heterostructure during hydrogen ab/de-sorption process. Additionally, the typical doublets located at 456.8 and 462.5 eV of Ti^3+^ for as-synthesized MgH_2_/TiO_2_ composites further confirm the successful introduction of oxygen vacancies. Moreover, it can be seen that the Ti 2*p* signals of Ti^2+^ appear in the dehydrogenated samples and Ti^2+^ peaks shift to higher binding energies in the re-hydrogenated samples. The presence of Ti^2+^ peaks testifies the formation of low-valence Ti-H active species. The slight change of the peak position of Ti^2+^ demonstrates that Ti-H active species could act as the intermediates to promote the transportation of electrons and hydrogen [19], further proving that the Mg-Ti oxide acts as a pathway to promote hydrogen diffusion. It is well known that the electronegativity of Ti (1.54) is between Mg (1.31) and H (2.2) [21, 54]. Variable valence of Ti could also facilitate the electron transfer between Mg^2+^ and H^−^, providing a favorable condition for weakening the Mg-H bond. In addition, numerous multi-phase interfaces composed of MgH_2_ and multivalence Ti promote the diffusion of hydrogen and electron and provide more nucleation sites for MgH_2_/Mg. Thus, the existence of multivalence Ti-based catalysts accounts for the significantly enhanced hydrogen storage performances of the MgH_2_/TiO_2_ heterostructure.

Based on the experimental results and analyses, it is concluded that the superior hydrogen storage performances of the MgH_2_/TiO_2_ heterostructure can be attributed to the intrinsic and extrinsic synergistic effects, as illustrated in Fig. 6e: (1) Graphene-like 2D TiO_2_ nanosheets with a high specific surface area provide the unimpeded channels for the rapid diffusion of hydrogen and more nucleation sites for MgH_2_/Mg, thereby improving the kinetics of de/re-hydrogenation. (2) Nano-sized MgH_2_ are uniformly confined on TiO_2_ nanosheets to form a flower-like heterostructure, which significantly suppresses the growth and agglomeration of MgH_2_ during the hydrogen ab/de-sorption processes, ensuring the excellent cycling stability. Meanwhile, the re-stacking of TiO_2_ nanosheets can be hampered in turn. (3) Abundant oxygen vacancies introduced by the "one-stone-two-birds" strategy significantly enhance the electrical conductivity of TiO_2_ NS and provide additional active sites for transportation of electrons and hydrogen, leading to greatly enhanced hydrogen sorption kinetics. (4) The ternary Mg-Ti oxide catalyst located at the interface of MgH_2_ and TiO_2_ acts as the pathway to facilitate hydrogen diffusion, resulting in improved hydrogen storage properties. In addition, Mg-Ti oxide possesses better ductility and flexibility than pure MgO. Thus, due to the stable interfacial bonding strength between oxide layers and the MgH_2_ nanoparticles, Mg-Ti oxide may exhibit a long-term confinement effect on MgH_2_ nanoparticles and significantly buffer successive expansion and contraction of MgH_2_ particles during de/re-hydrogenation cycles, improving reversible cycling performance. (5) Multi-valence Ti-based catalysts favor the electron transfer between Mg^2+^ and H^−^, weakening the Mg-H bond. Additionally, numerous multi-phase interfaces composed of Mg^2+^ and multi-valence Ti containing species could provide more diffusion pathways for hydrogen and more nucleation sites for MgH_2_/Mg.

## Conclusion

In the present work, a flower-like MgH_2_/TiO_2_ heterostructure has been successfully synthesized using oxygen vacancy-rich 2D TiO_2_ NS as the functional scaffold. Benefitting from the intrinsic (nano-sized MgH_2_) and extrinsic (catalytic activity of Mg-Ti oxide at interfaces) effects as well as the incorporation of oxygen vacancies, the prepared MgH_2_/TiO_2_ heterostructure exhibits superior hydrogen storage performances. Specifically, the composite shows excellent desorption performances with a low onset desorption temperature down to 180 °C and a rapid initial dehydrogenation rate at 300 °C (2.116 wt% min^−1^), surpassing those of the state-of-the-art MgH_2_ systems catalyzed by TiO_2_. Meanwhile, the MgH_2_/TiO_2_ heterostructure displays an excellent cycling stability with a capacity retention of 98.5% after 100 cycles at 300 °C. This work offers a promising approach for applying graphene analogues to the construction of advanced hydrogen storage materials with 0D/2D heterostructures.

## Supplementary Information

Below is the link to the electronic supplementary material.

Supplementary file1 (DOCX 4837 KB)
