# Supplementary material for: Oxygen Vacancy-Rich 2D TiO2 Nanosheets: A Bridge Toward High Stability and Rapid Hydrogen Storage Kinetics of Nano-Confined MgH2
Source: Nanomicro Lett. 2022 Jul 15;14:144. doi: 10.1007/s40820-022-00891-9 (PMC9287516; doi:10.1007/s40820-022-00891-9)
Supplement: Supplementary file 1 — Supplementary file1 (DOCX 4837 KB) [file 40820_2022_891_MOESM1_ESM.docx]

Supporting Information for

**Oxygen Vacancy-Rich 2D TiO_2_ Nanosheets: A Bridge Toward High Stability and Rapid Hydrogen Storage Kinetics of Nano-Confined MgH_2_**

Li Ren^1,2,3^, Wen Zhu^1^, Yinghui Li^1^, Xi Lin^1^, Hao Xu^1^, Fengzhan Sun^1^, Chong Lu^4^, and Jianxin Zou^1,2,3^*

^1^National Engineering Research Center of Light Alloys Net Forming & State Key Laboratory of Metal Matrix Composites, Shanghai Jiao Tong University, Shanghai, 200240, PR China

^2^Shanghai Engineering Research Center of Mg Materials and Applications & School of Materials Science and Engineering, Shanghai Jiao Tong University, Shanghai, 200240, PR China

^3^Center of Hydrogen Science, Shanghai Jiao Tong University, Shanghai, 200240, PR China

^4^Instrumental Analysis Center of SJTU, Shanghai Jiao Tong University, Shanghai, 200240, PR China

*Corresponding author: E-mail: zoujx@sjtu.edu.cn; Tel: +86 21 54742381; Fax: +86 21 34203730

**Supplementary Figures and Tables**

**Table S1** Summary of the precursors used for the synthesis of MgH_2_/TiO_2_ heterostructure

| Samples | Amount of MgBu_2_ (mL) | Amount of TiO_2_ (mg) |
| --- | --- | --- |
| blank MgH_2_ | 1.8 | 0 |
| 40MgH_2_/TiO_2_ | 0.8 | 30 |
| 50MgH_2_/TiO_2_ | 1.2 | 30 |
| 60MgH_2_/TiO_2_ | 1.8 | 30 |
| 70MgH_2_/TiO_2_ | 2.7 | 30 |
| 80MgH_2_/TiO_2_ | 4.6 | 30 |

Note:

The amounts of MgH_2_ in the prepared MgH_2_/TiO_2_ heterostructures are calculated according to the formula below:

MgBu_2_ + H_2_ → MgH_2_ + C_4_H_10_

For example, 1.8 mL of MgBu_2_ (1.8 mmol) can be hydrogenated to obtain 46.8 mg of MgH_2_. Therefore, the weight percentage of MgH_2_ in the MgH_2_/TiO_2_ heterostructure (m(TiO_2_) = 30 mg) is calculated to be about 60 wt.% (46.8 mg/(30+46.8) mg).

**
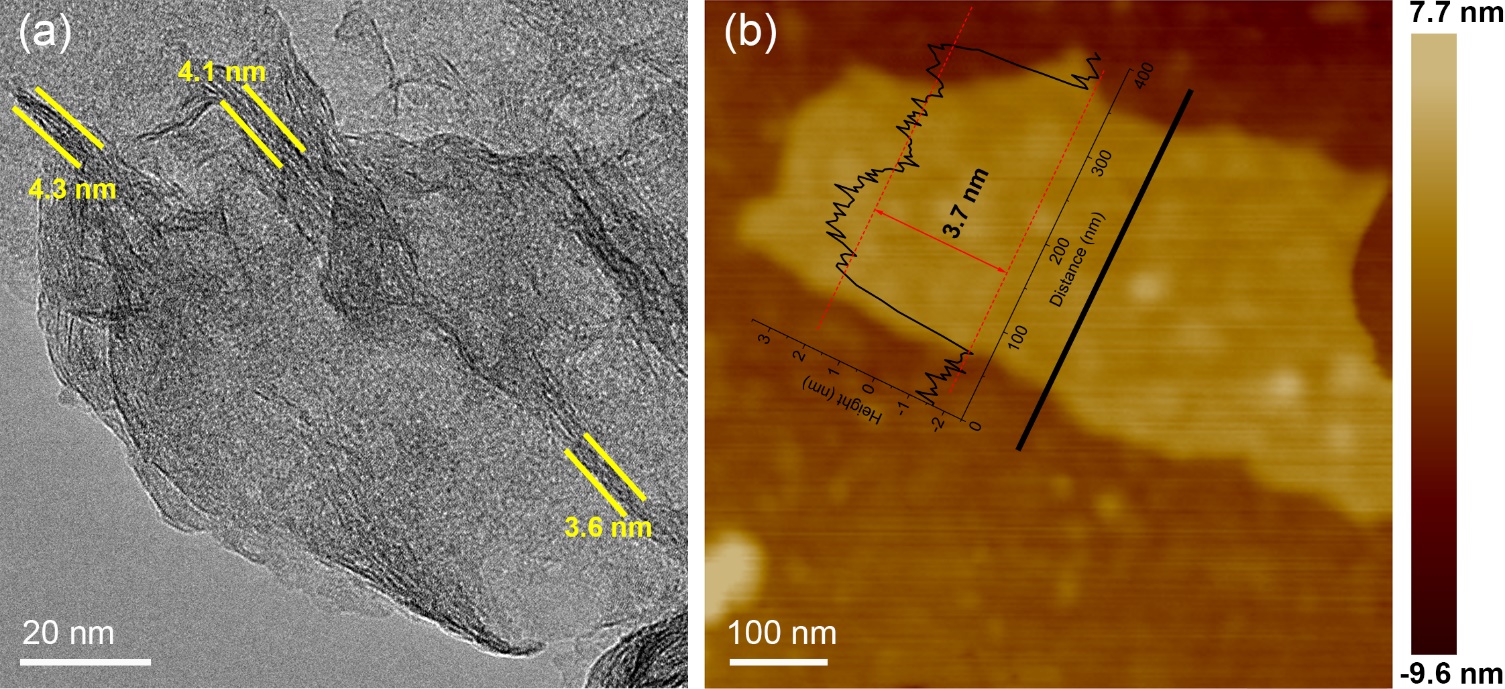
**

**Fig. S1** **a** Typical TEM image showing the edge configuration of 2D TiO_2_ NS. **b** AFM image demonstrating the thicknesses of a 2D TiO_2_ nanosheet to be ~3.7 nm

**
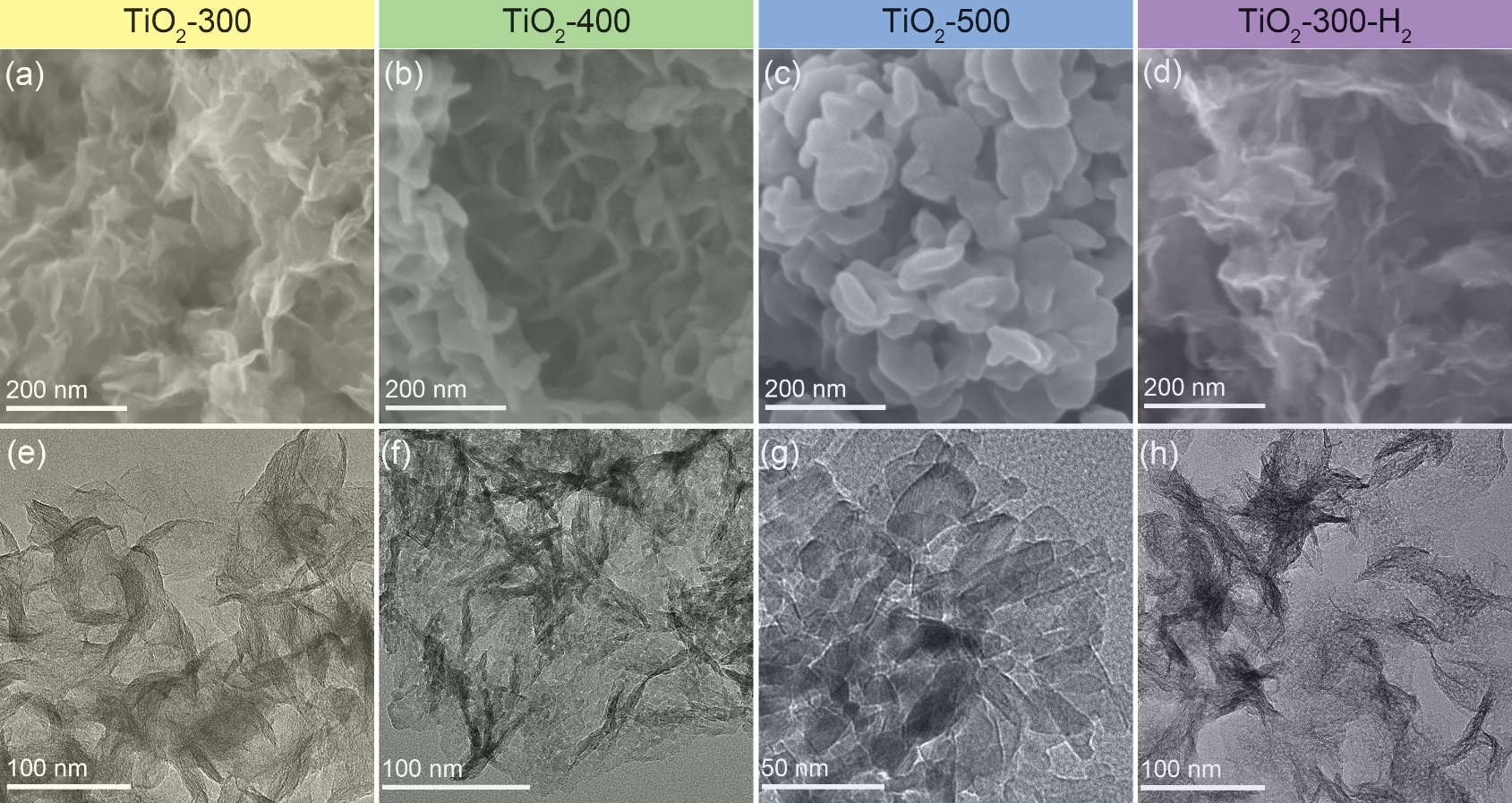
**

**Fig. S2** Typical SEM images **a-d** and TEM images **e-h** of TiO_2_ NS annealed at different temperatures

**
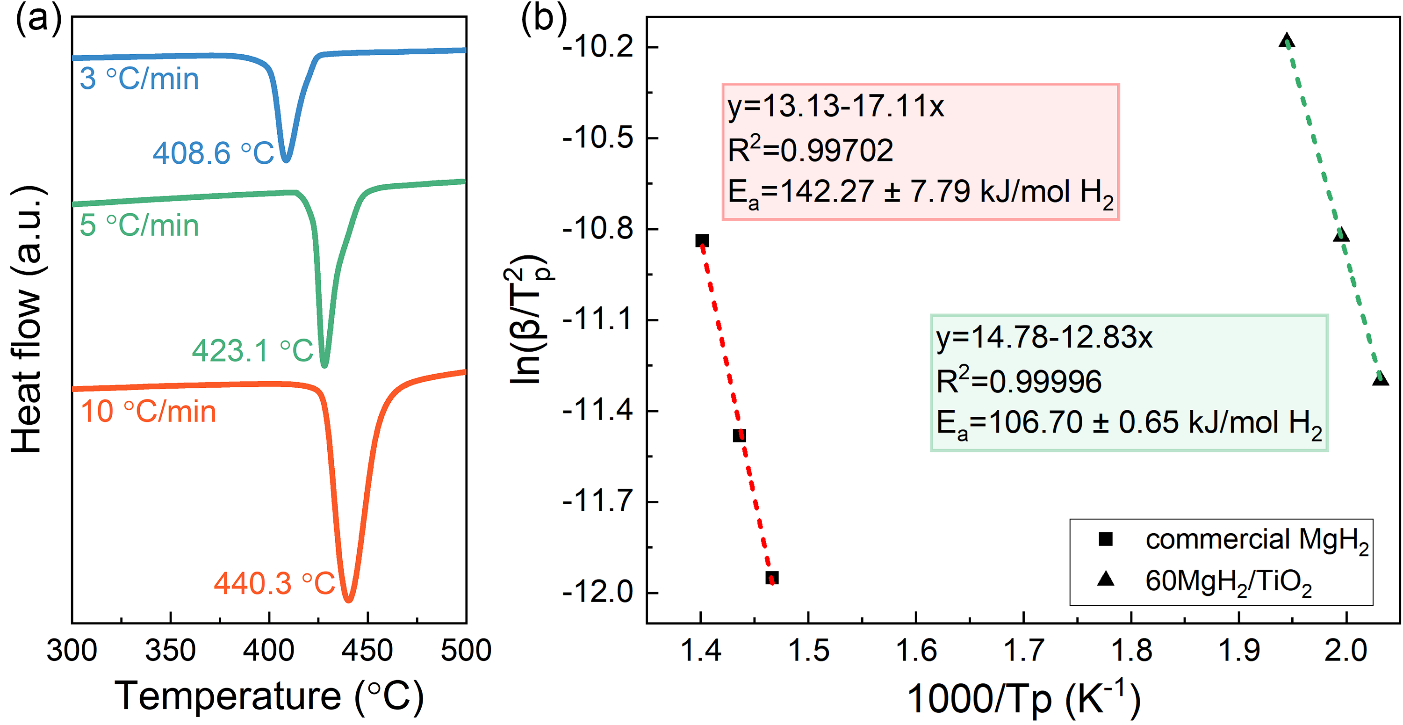
**

**Fig. S3** **a** DSC curves of the commercial MgH_2_. **b** Kissinger’s plots of 60MgH_2_/TiO_2_ and commercial MgH_2_

**
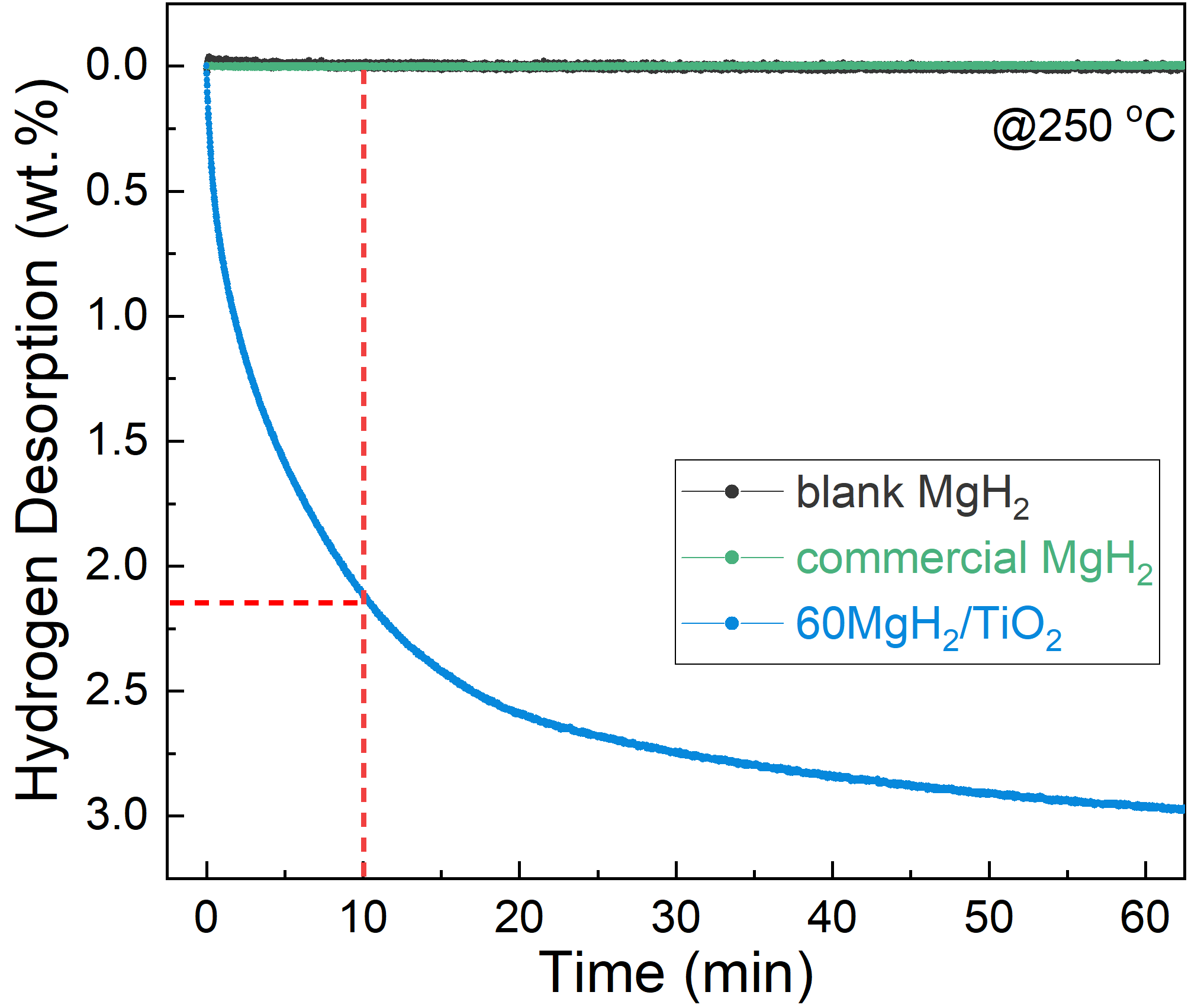
**

**Fig. S4** The comparison of isothermal desorption behaviors of blank MgH_2_, commercial MgH_2_, and 60MgH_2_/TiO_2_


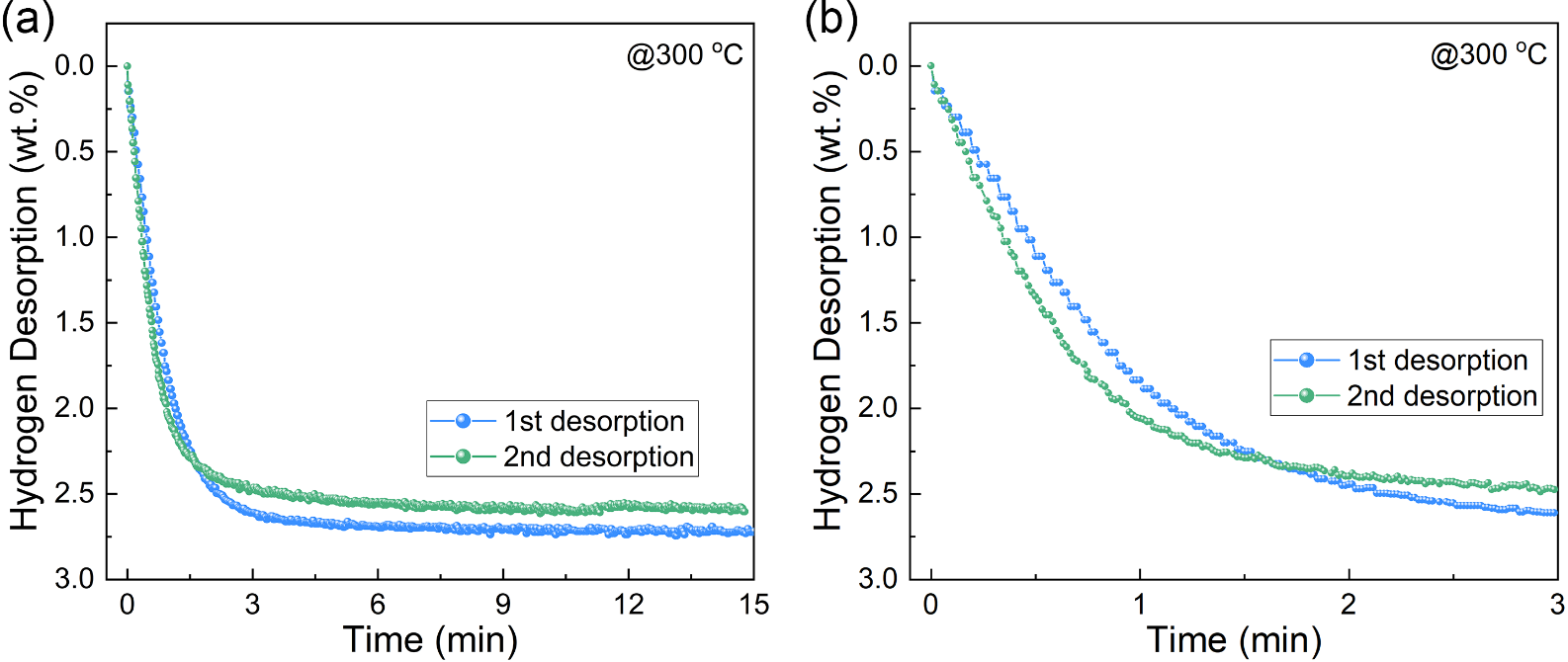


**Fig. S5 a** The comparison of isothermal desorption behaviors of 60MgH_2_/TiO_2_ in the first two cycles at 300 °C and **b** the corresponding enlarged view for the first three minutes


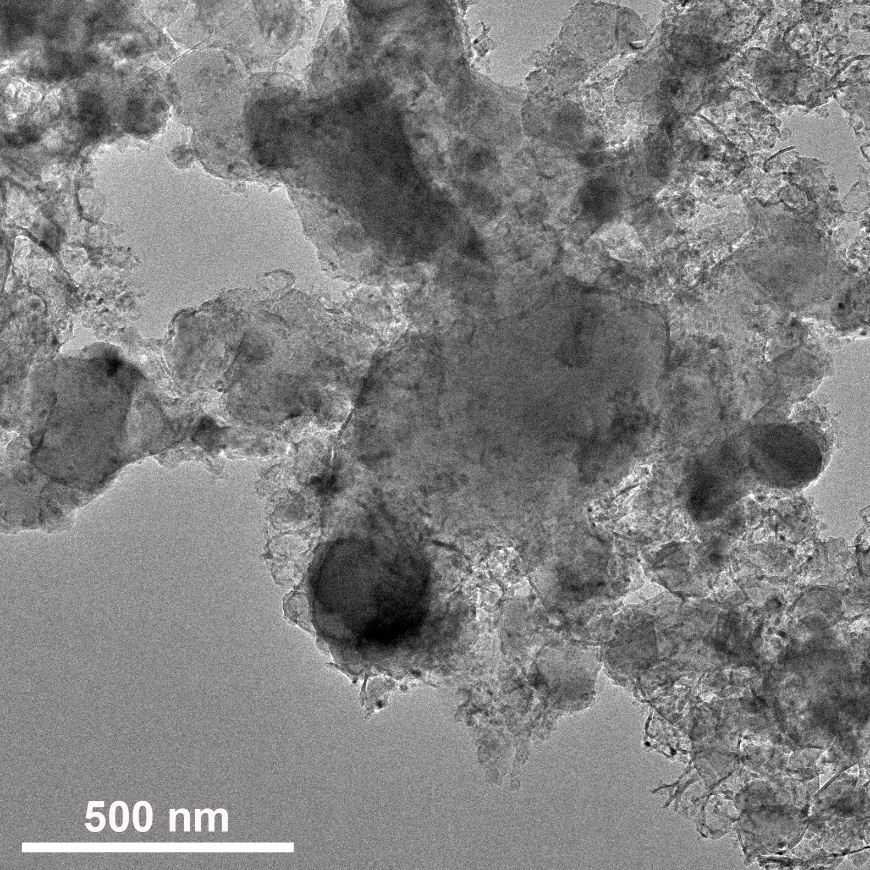


**Fig. S6** Typical TEM image of blank MgH_2_ synthesized without TiO_2_ NS after several de/re-hydrogenation cycles


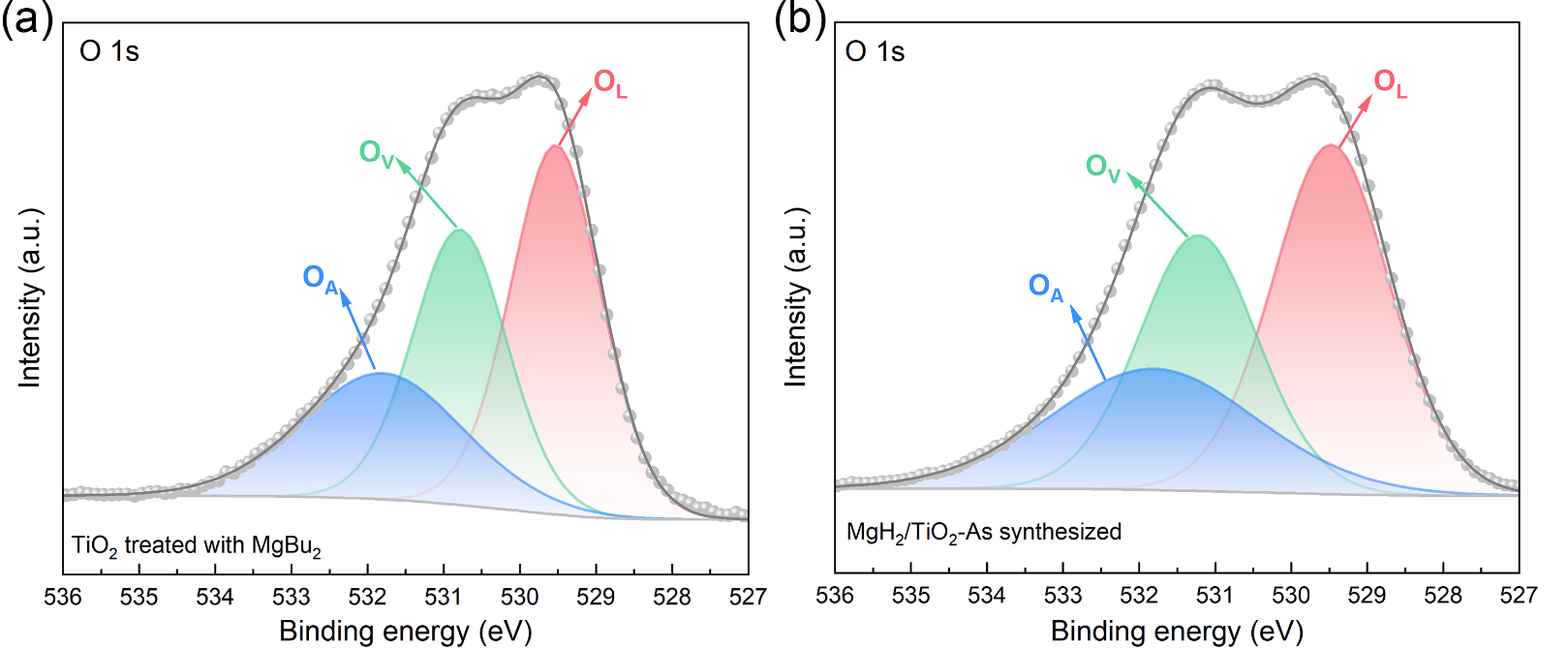


**Fig. S7** O 1s XPS spectra of **a** the TiO_2_ nanosheets treated with MgBu_2_ and **b** the as-synthesized MgH_2_/TiO_2_ heterostructure (O_A_: Adsorbed oxygen; O_V_: Oxygen vacancy; O_L_: Lattice oxygen)


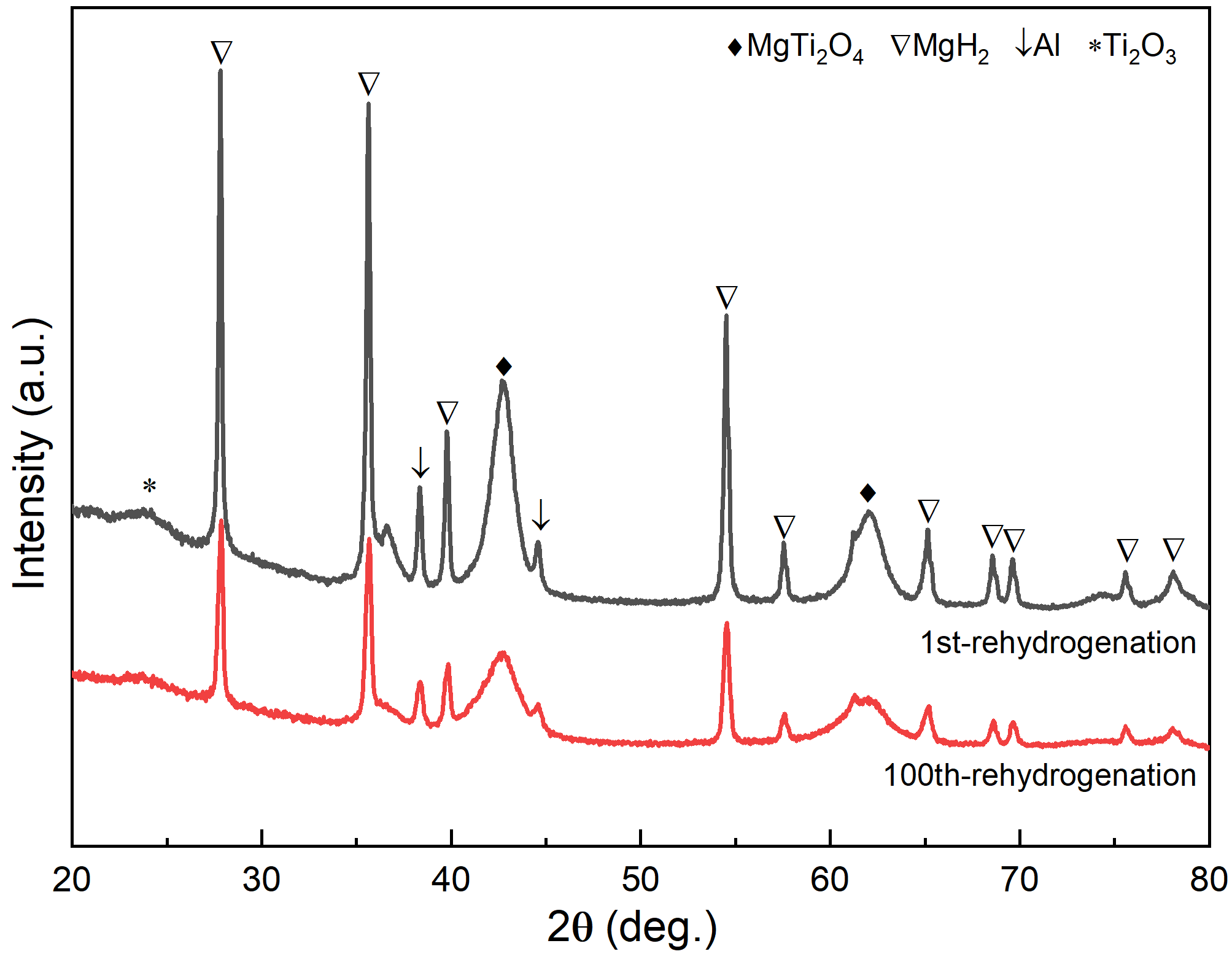


**Fig. S8** XRD patterns of the re-hydrogenated MgH_2_/TiO_2_ heterostructure after 1 de/re-hydrogenation cycle and 100 de/re-hydrogenation cycles


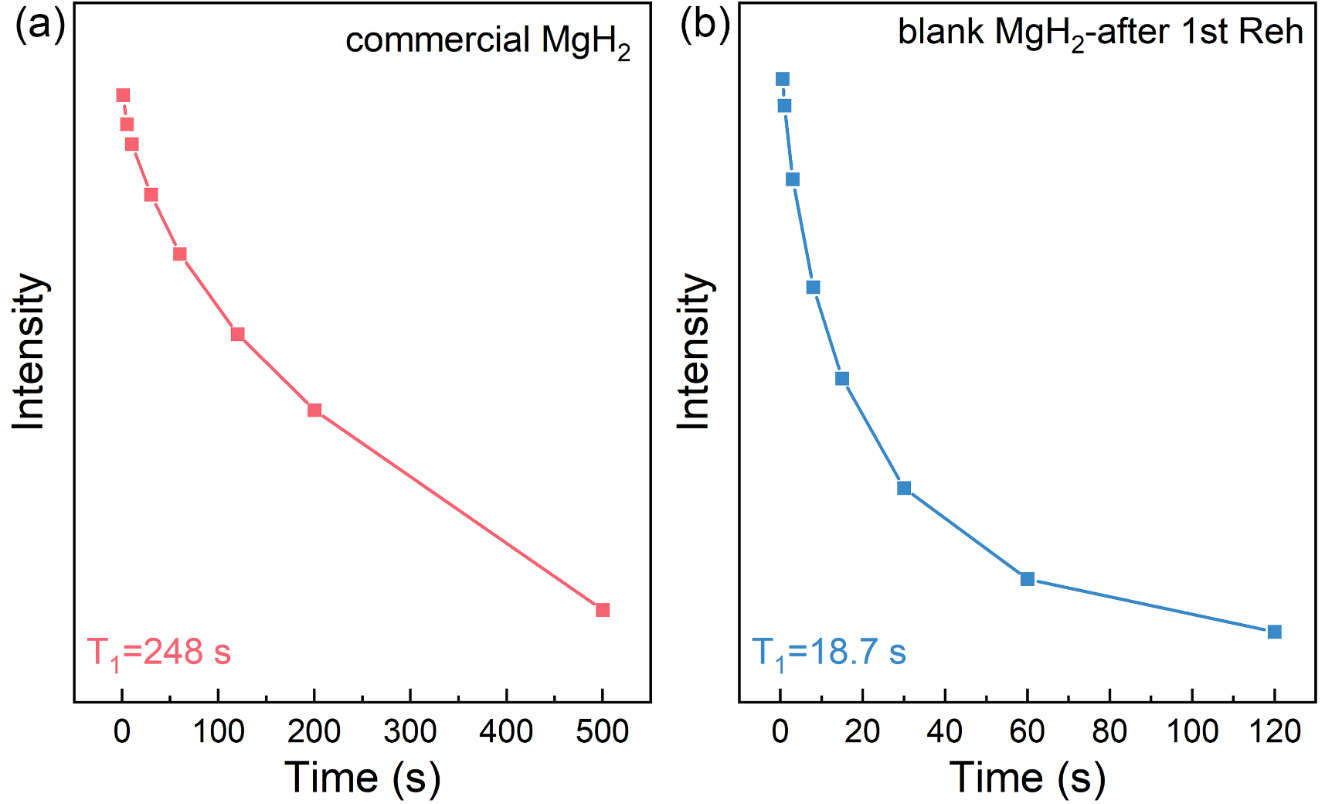


**Fig. S9** NMR spin-lattice relaxation curves of **a** commercial MgH_2_ and **b** blank MgH_2_ after the 1st re-hydrogenation


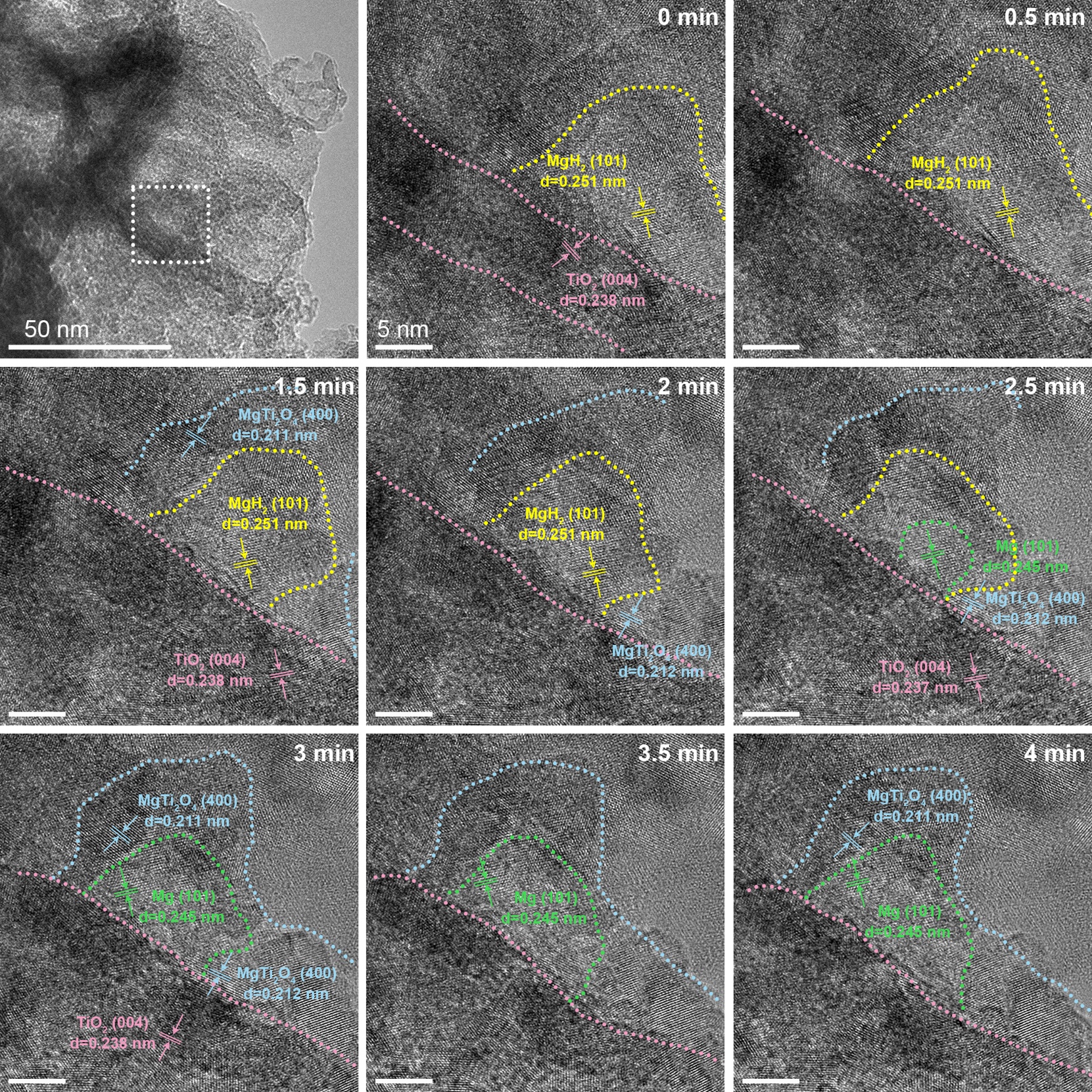


**Fig. S10** Typical TEM and HRTEM images of the hydrogenated MgH_2_/TiO_2_ composites under electron beam radiation during the hydrogen desorption process


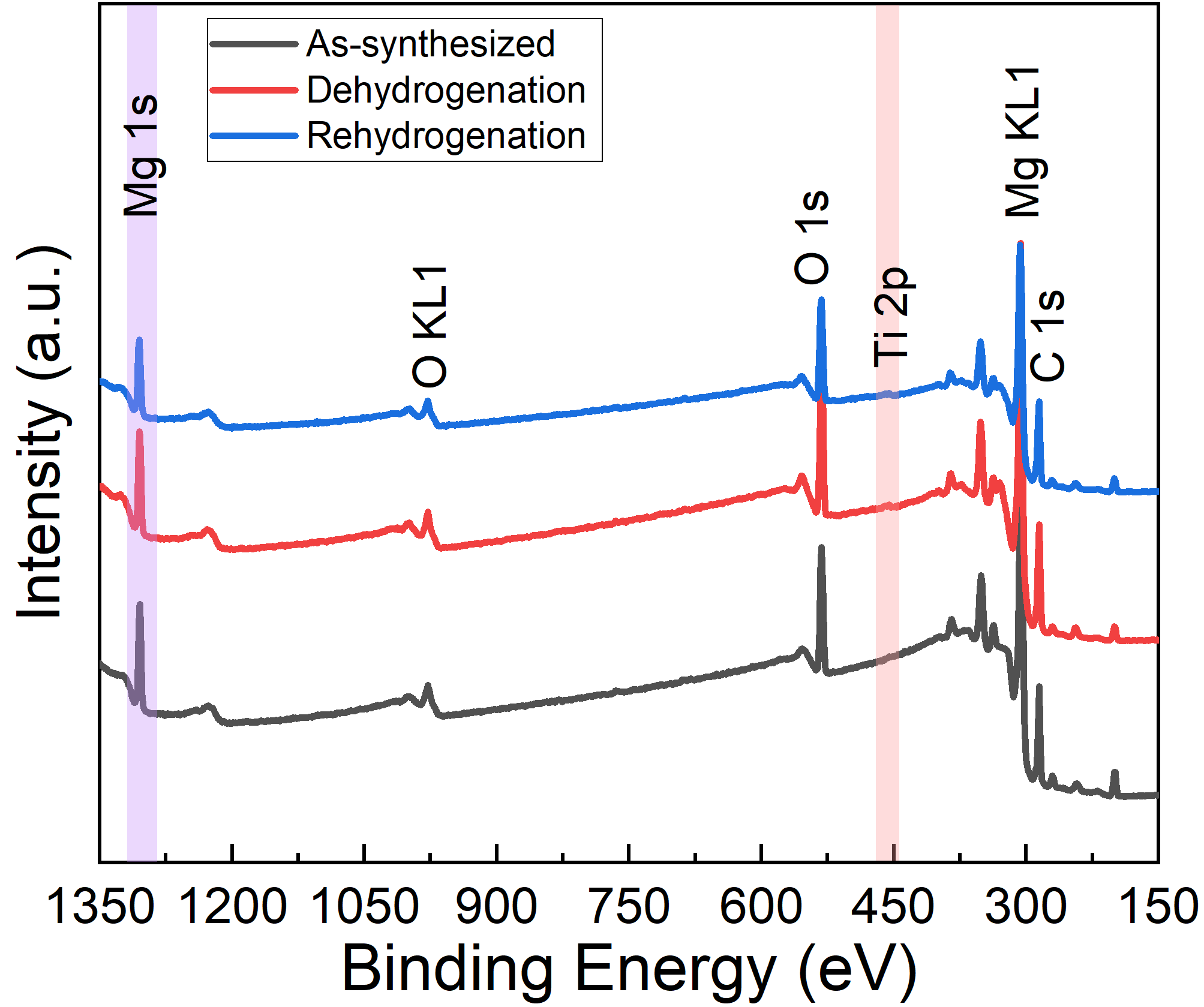


**Fig. S11** XPS spectra of the MgH_2_/TiO_2_ heterostructure at different states

**Table S2** Comparison of hydrogen storage performances of MgH_2_/TiO_2_ heterostructure in the present work with other MgH_2_ based systems

| Scaffolds /catalysts ^a)^ | Loading capacity (wt.%) | System gravimetric capacity (wt.%) | T_onset_ (^o^C) (hydrogen desorption) | T_peak_ (^o^C) (heating rate: 5 ^o^C min^-1^) | Initial dehydrogenation rate at 300 ^o^C (wt% min^-1^) | | Refs |
| --- | --- | --- | --- | --- | --- | --- | --- |
| Graphene | 60 | 4.5 | 250 | 340 | 0.7 | [S1] | |
| CMK-3 | 40-60 | 2.4-3.7 | 250 | 300-260 | None | [S2] | |
| Carbon aerogels | 10 | 1.5 ^b)^ | 220 | 280 | None | [S3] | |
| TiO_2_@C ^a)^ | 90 | 6.5 | 205 | 259 (6 ^o^C min^-1^) | 1.12 | [S4] | |
| Carbon aerogels | 18.2 | 1.14 | 200 | 385 (3.6 ^o^C min^-1^) | None | [S5] | |
| 3D TiO_2_ ^a)^ | 95 | 6.7 | 199.2 | 245.4 | None | [S6] | |
| 2D TiO_2_ (B) ^a)^ | 90 | 6.29 | 200 | 227.6 | None | [S7] | |
| BCNT | 78 | 5.79 | 237.5 | 276.7 | 0.75 | [S8] | |
| TiO_2_@Gr ^a)^ | 90 | 6.5 | 270 | 290 (10 ^o^C min^-1^) | 0.38 | [S9] | |
| CoS | 60 | 3.1 | 297 | 314 | 0.255 | [S10] | |
| Ni-MOF | 50 | 2.7 | 314.5 | 330.4 | None | [S11] | |
| TiO_2_ | 60 | 3.4 | 180 | 228.7 | 2.116 | This work | |

Note:

^a)^ The synthesis method is high-energy ball milling.

^b)^ Calculation of the gravimetric capacity does not include the weight of the scaffold.

**Supplementary References**

[S1] Y. Huang, G. Xia, J. Chen, B. Zhang, Q. Li, X. Yu, One-step uniform growth of magnesium hydride nanoparticles on graphene. Prog. Nat. Sci. **27**(1), 81-87 (2017). <https://doi.org/10.1016/j.pnsc.2016.12.015>

[S2] M. Konarova, A. Tanksale, J. Norberto Beltramini, G. Qing Lu, Effects of nano-confinement on the hydrogen desorption properties of MgH_2_. Nano Energy **2**(1), 98-104 (2013). <https://doi.org/10.1016/j.nanoen.2012.07.024>

[S3] Y. S. Au, M. K. Obbink, S. Srinivasan, P. C. M. M. Magusin, K. P. de Jong, P. E. de Jongh, The Size Dependence of Hydrogen Mobility and Sorption Kinetics for Carbon-Supported MgH_2_ Particles. Adv. Funct. Mater. **24**(23), 3604-3611 (2014). <https://doi.org/10.1002/adfm.201304060>

[S4] X. Zhang, Z. Leng, M. Gao, J. Hu, F. Du, J. Yao, H. Pan, Y. Liu, Enhanced hydrogen storage properties of MgH_2_ catalyzed with carbon-supported nanocrystalline TiO_2_. J. Power Sources **398**, 183-192 (2018). <https://doi.org/10.1016/j.jpowsour.2018.07.072>

[S5] T. K. Nielsen, K. Manickam, M. Hirscher, F. Besenbacher, T. R. Jensen, Confinement of MgH_2_ Nanoclusters within Nanoporous Aerogel Scaffold Materials. ACS Nano **3**(11), 3521-3528 (2009). <https://doi.org/10.1021/nn901072w>

[S6] M. Zhang, X. Xiao, B. Luo, M. Liu, M. Chen, L. Chen, Superior de/hydrogenation performances of MgH_2_ catalyzed by 3D flower-like TiO_2_@C nanostructures. J. Energy Chem. **46**, 191-198 (2020). <https://doi.org/10.1016/j.jechem.2019.11.010>

[S7] M. Chen, X. Z. Xiao, M. Zhang, J. F. Mao, J. G. Zheng, M. J. Liu, X. C. Wang, L. X. Chen, Insights into 2D graphene-like TiO_2_ (B) nanosheets as highly efficient catalyst for improved low-temperature hydrogen storage properties of MgH_2_. Mater. Today Energy **16**, 100411 (2020). <https://doi.org/10.1016/j.mtener.2020.100411>

[S8] M. Liu, S. Zhao, X. Xiao, M. Chen, C. Sun, Z. Yao, Z. Hu, L. Chen, Novel 1D carbon nanotubes uniformly wrapped nanoscale MgH_2_ for efficient hydrogen storage cycling performances with extreme high gravimetric and volumetric capacities. Nano Energy **61**, 540-549 (2019). <https://doi.org/10.1016/j.nanoen.2019.04.094>

[S9] S. K. Verma, A. Bhatnagar, V. Shukla, P. K. Soni, A. P. Pandey, T. P. Yadav, O. N. Srivastava, Multiple improvements of hydrogen sorption and their mechanism for MgH_2_ catalyzed through TiH_2_@Gr. Int. J. Hydrog. Energy **45**(38), 19516-19530 (2020). <https://doi.org/10.1016/j.ijhydene.2020.05.031>

[S10] Z. Ma, S. Panda, Q. Zhang, F. Sun, D. Khan, W. Ding, J. Zou, Improving hydrogen sorption performances of MgH_2_ through nanoconfinement in a mesoporous CoS nano-boxes scaffold. Chem. Eng. J. **406**, 126790 (2021). <https://doi.org/10.1016/j.cej.2020.126790>

[S11] Z. Ma, Q. Zhang, S. Panda, W. Zhu, F. Sun, D. Khan, J. Dong, W. Ding, J. Zou, In situ catalyzed and nanoconfined magnesium hydride nanocrystals in a Ni-MOF scaffold for hydrogen storage. Sustain. Energy Fuels **4**(9), 4694-4703 (2020). <https://doi.org/10.1039/D0SE00818D>
